# Transport Barriers Influence the Activation of Anti‐Tumor Immunity: A Systems Biology Analysis

**DOI:** 10.1002/advs.202304076

**Published:** 2023-11-10

**Authors:** Mohammad R. Nikmaneshi, James W. Baish, Hengbo Zhou, Timothy P. Padera, Lance L. Munn

**Affiliations:** ^1^ Department of Radiation Oncology Massachusetts General Hospital and Harvard Medical School Boston MA 02114 USA; ^2^ Biomedical Engineering Bucknell University Lewisburg PA 17837 USA

**Keywords:** anti‐tumor immunity, immune activation, radiotherapy, T cell trafficking, tumor‐induced antigen

## Abstract

Effective anti‐cancer immune responses require activation of one or more naïve T cells. If the correct naïve T cell encounters its cognate antigen presented by an antigen presenting cell, then the T cell can activate and proliferate. Here, mathematical modeling is used to explore the possibility that immune activation in lymph nodes is a rate‐limiting step in anti‐cancer immunity and can affect response rates to immune checkpoint therapy. The model provides a mechanistic framework for optimizing cancer immunotherapy and developing testable solutions to unleash anti‐tumor immune responses for more patients with cancer. The results show that antigen production rate and trafficking of naïve T cells into the lymph nodes are key parameters and that treatments designed to enhance tumor antigen production can improve immune checkpoint therapies. The model underscores the potential of radiation therapy in augmenting tumor immunogenicity and neoantigen production for improved ICB therapy, while emphasizing the need for careful consideration in cases where antigen levels are already sufficient to avoid compromising the immune response.

## Graphical abstract

To mount an immune response against a tumor, naïve T cells need to be activated by an antigen produced by the tumor. Here, mathematical modeling is used to explore the possibility that immune activation is prevented or delayed due to transport limitations that prevent the colocalization of cognate naïve T cells and tumor antigen in the same lymph node.

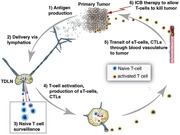

## 1 Introduction

Immune checkpoint blockers (ICBs) have transformed cancer treatment by inhibiting the interactions between receptor‐ligand pairs that limit the immune response—so‐called immune checkpoints, including CTLA‐4 and PD‐1/PD‐L1.^[^ ^1^, ^2^ ^]^ ICB therapy has produced durable responses in patients with advanced melanoma and non‐small cell lung cancer, among other cancers.^[^ ^3^, ^4^ ^]^ Although extremely encouraging, not all patients benefit, and a better understanding of immune function and checkpoint control is needed to improve outcomes.^[^ ^5^ ^]^

Many processes are involved in initiating and sustaining anti‐cancer immune responses, including the generation of antigen and its presentation on major histocompatibility complex (MHC) molecules. A less‐studied and potentially critical step is the physical colocalization of cancer specific naïve T‐cells (nTcells) with antigen presenting cells (APCs) displaying their cognate antigen. This step is necessary for the generation of anti‐tumor effector T cells and is thought to take place primarily in lymph nodes.^[^ ^6^, ^7^, ^8^, ^9^ ^]^ T‐cell activation occurs when an nT‐cell encounters an APC (e.g., dendritic cell) displaying antigen on MHC molecules that the nT‐cell can specifically recognize via its T‐cell receptor. This T‐cell receptor/MHC/cognate antigen interaction (signal 1) needs to also have co‐stimulatory signaling between the nT‐cell and APC (signal 2) to induce the activation of effector T‐cell responses.

The co‐localization of the APC, nT‐cell and cognate antigen is facilitated by the lymphatic system. As initial lymphatic vessels absorb interstitial fluid, they also collect antigen and APCs. Both are then concentrated in lymph nodes, creating a target‐rich environment for nT‐cells to surveil.^[^ ^10^ ^]^

Although there are ≈10^11^ nT‐cells in humans, each clone has unique specificity.^[^ ^11^ ^]^ Because humans need to harbor a large variety of nT‐cell clones to protect against a diversity of potential pathogens, there are few nT‐cells specific for any given antigen.^[^ ^12^, ^13^, ^14^, ^15^ ^]^ This may limit immune activation, which requires the colocalization of the correct nT‐cell with sufficient cognate antigen.^[^ ^11^ ^]^ For activation of anti‐tumor immunity, one of the few nT‐cells capable of recognizing tumor antigen needs to visit a lymph node where tumor antigen has accumulated and the microenvironment is not already immune suppressive. Cancer‐specific nT‐cells execute a random search throughout the body, sampling secondary lymphoid organs. Thus, a nT‐cell may circulate many times before finding a lymph node, and many more times before entering a lymph node with sufficient cognate antigen. As there are multiple parallel paths through the circulation that an nT‐cell could take, it becomes a probabilistic process to bring a cognate nT‐cell to a lymph node draining the tumor.^[^ ^16^ ^]^ Once activated, the expanding population of clonal T cells leaves the node, enters the blood, and travels through the blood circulation. After adhesion to blood vessel endothelium and extravasation, they can enter the target tissue and kill antigen‐bearing cells unless checkpoint molecules (e.g., PD‐1) prevent it (which can result in anergy or “exhaustion” of the effector T‐cell).

The probabilistic nature of nT‐cell sampling of lymph nodes makes it challenging to predict when the nT‐cell will arrive in a tumor draining lymph node (TDLN)—and whether there will be sufficient antigen when it arrives. Activation of nT‐cells also requires that the LN with antigen is not immunosuppressed when the cognate nT‐cell arrives. Such stochastic processes may explain why previously‐validated biomarkers do not predict with certainty the response of a given patient to ICB therapy.^[^ ^17^, ^18^ ^]^ Randomness may also explain why genetically identical mice can have different tumor responses to the same ICB therapy.^[^ ^19^, ^20^ ^]^ For example, ≈40% of mice with identical E0771 tumors growing in the mammary fat pad respond to anti‐PD‐1 therapy, while tumors in the remaining 60% of mice continue to grow like controls.^[^ ^20^ ^]^ Because these mice are genetically identical, the binary response suggests that there is a stochastic process that leads to immune activation in only some mice. Furthermore, when two tumors are grown in different mammary fat pads in the same mouse, they either both respond to the ICB or they both continue to grow like control tumors.^[^ ^20^ ^]^ This suggests that the “switch” that is activated in the responders operates at the systemic level rather than locally within a tumor. Interestingly, rechallenging long‐term surviving mice with the original cancer cells or a tumor harvested from a non‐responding mouse results in the rejection of the tumor.^[^ ^20^ ^]^ This indicates that the successful ICB response is not due to the creation of unique neoantigens in the responders, but rather a systemic‐level activation event that is able to recognize the original tumor antigens.

Here, we explore the hypothesis that anti‐cancer immune responses are limited by the frequency of encounters between naïve T cells and appropriate antigen/APCs using a pharmacokinetic‐based systems biology computational model. Such models represent each component of the system and how those components interact with one another over several length scales.^[^ ^21^, ^22^, ^23^, ^24^, ^25^, ^26^, ^27^, ^28^, ^29^, ^30^ ^]^ There is a rich literature of pharmacokinetic‐pharmacodynamic (PKPD) or quantitative systems pharmacology (QSP) models of the circulation of therapeutic agents, antigens, and immune cells in the blood and lymph.^[^ ^31^, ^32^, ^33^, ^34^ ^]^ The simplest of these consider only a single tumor and a single lymph node, while more detailed models include several additional organs. In addition, other models focus on the pumping dynamics of individual (or small networks of) lymphatic vessels that are responsible for lymph flow between the tumor (and peripheral tissues) and lymph nodes.^[^ ^35^, ^36^, ^37^, ^38^, ^39^, ^40^, ^41^ ^]^

**Figure 1:**
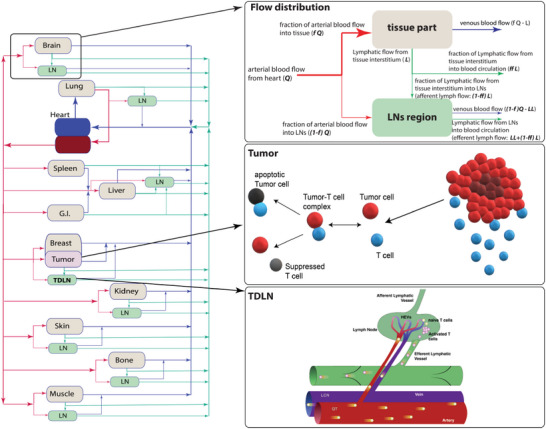
Schematics of the tumor‐immune microenvironments and the free diagram of the compartmental PKPD model. The arrows of blood and lymphatic flows are scaled corresponding with flow rates. Trafficking of T cells occurs through the systemic blood circulation, lymph node and exit through the efferent lymphatic vessel. In the flow distribution panel, *Q* is blood flow, *L* is lymphatic flow collected from tissue interstitium (afferent lymph), and *LL* is lymphatic flow collected from LN interstitium (efferent lymph). *f* is the fraction of blood flow into an organ tissue and *ff* is the fraction of tissue lymphatic flow, which is directly released into the systematic lymphatic flow. Thus, (1‐*f*)*Q* is the fractional flow rate of blood that enters a lymph node (LN‐FFR), and (1‐*ff*)*L* is the fraction of tissue lymphatic flow that enters a lymph node.

The whole‐body model includes compartments representing the tumor, lung, liver, spleen, intestine, brain, kidney, skin, bone, muscle, and heart (**Figure** **1**). Blood flow follows anatomically‐accurate pathways and is distributed to the various organs via branches in the arterial tree, according to flow volumes reported in the literature.^[^ ^26^, ^30^, ^42^ ^]^ Each compartment of the model contains a sub‐compartment for the organ and another sub‐compartment representing the lymph node(s) draining that organ. Each sub‐compartment receives a fraction of the blood flow entering that organ, and a fraction of interstitial fluid collected from the tissue is assumed to flow into the draining lymph nodes (LNs). Lymph exits the LNs and enters the converging network of lymphatic vessels to return to the venous circulation.

The model simulates immune responses as follows. We calculate tumor growth, antigen production, and the accumulation of antigen in various LNs. Antigen produced by the tumor either convects or is carried by APCs into lymphatic vessels and then accumulates in the tumor draining lymph node (TDLN). A fraction of the antigen (Table S1 of the supplementary material) also passes through the initial lymph node to arrive in additional LNs or return to the blood circulation. This simulates the bypass of antigen through the LN subcapsular sinus.^[^ ^43^ ^]^

For the circulation of the naïve T cells, we use a stochastic model to keep track of their location as they circulate through the blood stream, visiting various tissues and LNs during each pass through the blood system (see Figure S8, Supporting Information). At bifurcations, the direction of the naïve T cell is determined by a random function weighted by the relative flow rates in the daughter branches. The residence time in each lymph node is 10 h, and the residence times for the other organs are listed in Table S1 (Supporting Information).^[^ ^44^, ^45^ ^]^ We assume that any nT‐cell that enters the blood system of a lymph node will extravasate into the node via HECs. Once in the node, we use a threshold to determine whether the nT‐cell becomes activated based on the local level of antigen in that node.

If an nT‐cell is activated, it becomes a cytotoxic T lymphocyte (CTL) and starts to proliferate in the node. Exit of the CTLs from the node is determined by a Heaviside function based on a limited capacity of the LN to contain CTLs. CTLs that exit the LN enter the systemic circulation (through lymphatic drainage). They bind to activated tumor blood vessels and extravasate into the tumor tissue. Once they enter the tumor, they kill cancer cells if the immune checkpoint status is sufficiently permissive.

In the simulations, tumor volume is influenced by intrinsic growth of cancer cells and cancer cell killing by T cells or radiation therapy. T cell activation is affected by antigen accumulation in the lymph nodes and the antigen immunogenicity, which is related to the activation threshold.

## 2 Results and Discussion

**Figure 2:**
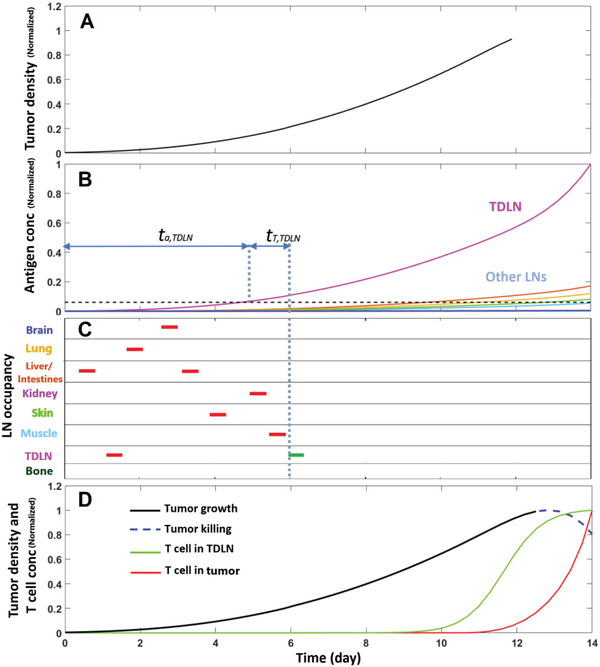
Simulating activation of anti‐tumor immunity. Shown are results from a single patient. A) Tumor growth normalized by maximum value of tumor density. B) tumor‐induced antigen accumulation in different lymph nodes, normalized by the maximum value of antigen concentration. C) stochastic circulation of naïve T cells in different lymph nodes. The colored lines show different LN sites and bars represent resident times of a naïve T cell in each LN. The liver/intestines LN represents abdominal LNs (LN_Abd). D) tumor growth, tumor killing by effector T cells, T cell accumulation in the TDLN and tumor—the values are normalized to the maximum value in each case.

To demonstrate the key features of the model, we first simulated a single patient with a tumor growing in the breast. Note that the location of the primary tumor affects the dissemination of antigen to various lymph nodes, so results will be somewhat affected by the site of tumor initiation. As the tumor grows (**Figure** **2**A), it begins to release antigen into the surrounding tissue, which is collected by lymphatic vessels and passed to the draining lymph node basin (represented by a single node for model simplicity). Some antigen can also bypass the tumor draining lymph node, entering the systemic circulation where it is diluted, but can potentially accumulate in other nodes more distant from the tumor (Figure 2B). The model assumes that no activation can take place until a threshold level of antigen is present in a node (dashed line, Figure 2B). At the same time, a naïve T cell circulates through the blood system, entering various LNs in a random search for antigen (Figure 2C). If it enters a LN with sufficient antigen, it becomes activated and starts to proliferate. The resulting effector T cells exit the node, infiltrate the tumor, and begin killing cancer cells (Figure 2D).

There are two characteristic time delays associated with the model—the time it takes for sufficient antigen to accumulate in a given node, *t_a_*, and the time it takes for a naïve T cell to find the node, once it has sufficient antigen*, t*_T_. The first is deterministic, and the second is stochastic. They are also not independent, as *t*_T_ depends on the accumulation rate of antigen and is relative to the time that antigen surpasses the threshold. In this particular simulation, antigen surpasses the threshold in the TDLN on day 5, and a nT‐cell visits that node 1 day later. This results in an anti‐tumor immune response that escalates starting at day 6 and decreases tumor volume by ≈20% between days 12 and 14.

### 2.1 Blood Flow Partitioning into Lymph Nodes (LNs) Affects Tumor‐Immune Response

One of the key events in immune surveillance is the entry of nT‐cells into the lymph node arterial supply. In the model, this step requires specification of the fractional flow rate of blood that enters a lymph node (LN‐FFR; (1‐*f*)*Q* in the Flow distribution panel of Figure 1). Because the experimental data are limited, we simulated a range of fractional flow rates into the LN, so that 0.5%, 1%, 5%, or 10% of nT‐cells were diverted to the LN vasculature in each organ.^[^ ^46^ ^]^ This is in the range of reported values as well as estimates based on relative tissue volumes (see Figure S1, Supporting Information). Another critical unknown parameter is the rate of production of antigen by the tumor. To examine the importance of antigen production, we simulated tumors with low, medium, and high antigen production rates.

**Figure 3:**
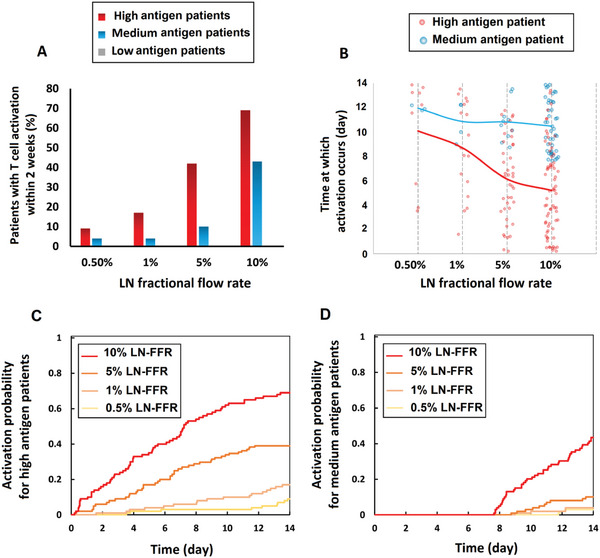
Effects of partitioning of nT‐cells into the LN circulation and antigen production rate on T cell activation. A) The probability of a single T cell activation event within 2 weeks of simulation for cancer patients with low, medium, and high tumor‐induced antigen rates under different LN fractional flow rates; 0.5%, 1%, 5%, and 10% of the total blood flow rate feeding each organ. B) Time elapsed before T cell activation in patients with high antigen rate (red circles) and medium antigen rate (blue circles); the averages are given by the red and blue lines, respectively. C) Cumulative event probability for T cell activation in high antigen patients, D) Cumulative event probability for T cell activation in medium antigen patients.

In the simulations, when sufficient antigen is encountered by a naïve T cell within a lymph node, the T cell becomes activated. We examined this process for cancer patients with low, medium, and high production rates of tumor antigen. Because of the stochastic nature of the model, we simulated 100 tumors in each group, which we followed for two weeks (**Figure** **3**A). The results show that increasing the fraction of nT‐cells that exit the blood circulation to enter the vasculature of a lymph node (LN‐FFR) dramatically enhances the probability of activation of nT‐cells in patients with high and medium—but not low—antigen production. The response in patients with high antigen production is even more sensitive to LN‐FFR than that in medium antigen patients.

We can also record the time at which activation occurs (Figure 3B). Increasing LN‐FFR can considerably decrease the time required for activation (*t*_a_ *+ t*_T_) in patients with high antigen production rate, but it has less impact on the time of patients with medium antigen production rate.

We then plotted the cumulative event probabilities for activation of the T cells for high (Figure 3C) and medium (Figure 3D) antigen production rates. These results show that increasing LN‐FFR or antigen production rate can decrease the time needed for T cell activation.

**Figure 4:**
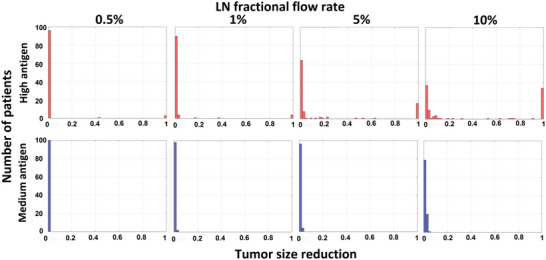
Tumor size reduction (TSR) ratio in response to activation of natural immunity with high (top) and medium (bottom) production rates of tumor‐induced antigen under different LN‐FFR. A TSR of zero indicates no response, while TSR = 1 indicates tumor eradication.

We next examined the ability of the activated T cells to reduce tumor volume. To simplify the biology, we assume that immune checkpoint inhibition is nearly complete and T cell exhaustion is minimal (relative rate constants = 0.99 and 0.01, respectively). Consequently, once the effector T cells circulate to the tumor, they are likely to initiate a sustained anti‐tumor response. With these assumptions, the key parameters that determine tumor eradication are the tumor growth rate and time of activation of the T cells. To examine tumor size reduction in the patients who had T cell activation, we calculated the relative tumor size reduction (TSR) (**Figure** **4**).

Considering only those patients with successful T cell activation in the 2‐week period, those with high antigen production had a much greater probability of tumor eradication than those with medium antigen production, due to the earlier activation of immunity. Data points between 0 and 1 indicate the fractional reduction in tumor volume relative to unchecked growth measured at the 2 week time point. These numbers are in the range reported for clinical treatment of melanoma and small cell lung cancer.^[^ ^47^, ^48^, ^49^, ^50^ ^]^ Increasing the LN‐FFR has a strong effect on the high antigen patients, but less effect on medium antigen production.

**Figure 5:**
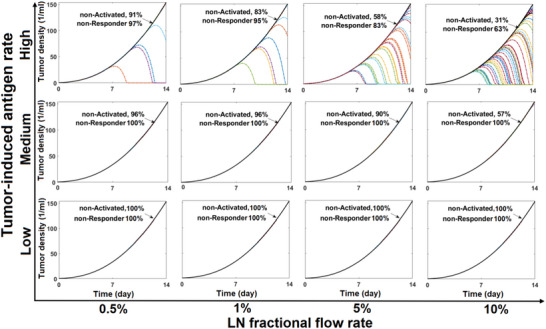
Effects of tumor antigen production rate and LN fractional flow rate (LN‐FFR) on T cell activation and tumor elimination. Each result shows tumor growth and the probabilities of non‐activated and non‐responder immunity of 100 cancer patients for two weeks. The black solid‐line shows non‐responder patients and colored dot‐dash lines show individual patients with tumor reduction due to T‐cell killing.

These results can also be visualized by plotting the tumor size as a function of time for each patient (**Figure** **5**). As explained in the supplementary material (Figure S2, Supporting Information), we used a simplified version of our model to estimate a threshold time for T cell activation. If the tumor is small enough when the effector T cells arrive, they can eliminate it; otherwise, tumor growth can out‐pace immune killing (Figure S2, Supporting Information). In this analysis, the critical time for nT‐cell activation is 4.62 days, and activation at this time leads to a tumor size reduction of 0.7 at the two‐week time point. If T cell activation takes longer than the threshold time, tumor growth outpaces the immune response in two weeks, and the tumor is not eradicated (designated “non‐responder” with TSR < 0.7). Earlier activation, before the threshold time, results in tumor clearance (“responder” with TSR within 0.7–1; see Figure S2, Supporting Information). In Figure 5, the plots show the time when anti‐tumor killing by the T cells is initiated and the time course of tumor shrinkage (dashed lines). For example, for the high antigen tumors and the case of 10% LN‐FFR, 69/100 patients had T cell activation within the two‐week period, and 37 of these occurred sufficiently early to decrease the tumor size by >70% (TSR range = 0.7–1, according to Figures 4 and 5, “High antigen patients, 10% LN fractional flow rate”). As expected, higher tumor antigen production rates increase the activation probability, and this is further enhanced by increasing LN‐FFR.

### 2.2 Can LN‐Sparing Radiation Therapy Enhance Anti‐Tumor Immunity?

We next examined how radiation therapy applied to the tumor and/or TDLN affects the activation of the immune response. There is evidence that radiation therapy can help generate neoantigens, thus synergizing with ICB therapies.^[^ ^32^, ^51^, ^52^ ^]^ On the other hand, it is thought that irradiation of a reactive TDLN can interrupt immune activation.^[^ ^53^ ^]^ To examine this with our model, we simulated irradiation of the tumor, which we assume kills cancer cells, any resident proliferating T cells and endothelial cells, and also increases effective antigen production rate due to antigen shedding from the irradiated cells. We also simulated irradiation of the TDLN, which we assume damages proliferating, activated T cells.

We simulated a 3‐week radiotherapy (RT) schedule, restricting the treatment to the tumor, the TDLN, or both. This was repeated for patients with low and high baseline antigen production to investigate the effects of RT on T cell activation and tumor killing. We assume that the RT doses are applied daily for five days each week. In this study, the TSR ratio is calculated based on the size of the non‐irradiated tumor at day 35 for RT applied to only the TDLN. For cases where the tumor is irradiated (with or without LN exposure), TSR is relative to the control case where there is no immune activation and tumor growth for 35 days.

**Figure 6:**
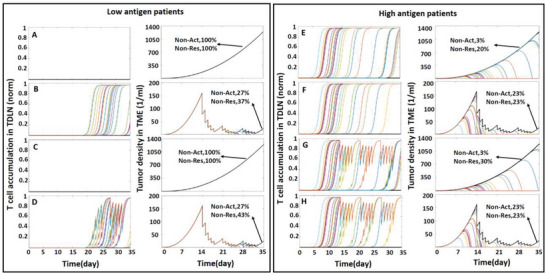
Effect of radiotherapy on tumor‐immune response in low and high antigen patients with LN‐FFR equal to 10%. Thirty cases were simulated for each condition. RT starts on day 14, with daily dose of 2 Gy, except two days (weekend), administered over three weeks. A) Non‐irradiated low antigen patients. B) RT on tumor of low antigen patients. C) radiation applied to the TDLN of low antigen patients. D) RT on tumor and TDLN of low antigen patients. E) non‐irradiated high antigen patients. F) RT on tumor of high antigen patients. G) RT to TDLN of high antigen patients. H) RT on tumor and TDLN of high antigen patient. The black line shows patients in which T‐cell activation does not occur. Colored lines show the T cell accumulation and tumor size in individual patients who have T‐cell activation. Tumor size and T cell numbers are stochastically increased by activation and are decreased by radiation treatment.

The results of T cell accumulation in the TDLN and tumor size are shown in **Figure** **6** for low and high antigen producing tumors. For the low antigen group, T cell activation only happens when RT is applied on the tumor or the tumor+TDLN (Figure 6A–D). Note that here, we assume that lymph node irradiation does not interfere with T cell activation—only proliferation/viability after activation. In both cases, 73% of patients have T cell activation within the 35‐day period (Figure 6B,D). The resulting immune response, combined with killing due to the radiation, leads to TSR‐classified responders in 63% and 57% of the tumor only and tumor+TDLN groups, respectively (Figure 6B,D).

For the high antigen group with no irradiation, 97% have activation within 35 days, and 80% are classified as responders (Figure 6E). Irradiating the tumor alone reduces both the T cell activation and responder probabilities to 77% (Figure 6F). Irradiation of the TDLN affects the responder probability more than the activation of T cells: with TDLN irradiation alone, 97% of patients have T cell activation, but only 70% have sufficient tumor killing by day 35 to fall into the “responder” category (Figure 6G). Interestingly, in these patients with high baseline antigen production, irradiating the TDLN with the tumor does not change the outcomes compared with irradiation of the tumor alone (Figure 6F,H).

The results of T‐cell accumulation in the TDLN for low and high antigen patients receiving RT to the tumor or TDLN or both are also shown in Figure 6. These results suggest that antigen production rate can be used to guide the appropriate timing of ICB therapy. Assuming that ICB therapy primarily affects killing at the tumor site, and not T cell activation in the nodes, it would be best to apply ICB drugs during the time window when T cell activation is most likely to be happening. This would allow the mobilized effector T cell to most efficiently kill the tumor. For low antigen patients irradiated on the tumor or on the tumor and TDLN, the optimal window for ICB would be days 19–30 (Figure 6B,D). The model predicts that patients with low antigen production and no tumor irradiation do not have T cell activation, and thus would not benefit from ICB therapy (Figure 6A,C). In contrast, most of the patients with high antigen production with or without RT have T cell activation and can benefit from ICB treatment starting as early as day 5. The results show that tumor irradiation in these patients with high baseline antigen production does not enhance T cell activation. To examine the effect of LN‐FFR on immunity and radiation response, we recalculated the results of Figure 6 for LN‐FFRs of 5%, 1%, and 0.5%. These results are shown in Figures S3–S5 (Supporting Information). To elucidate the importance of ICB for the patients with activated immune responses (Figure 6E), we compared RT applied to the tumor, ICB therapy and their combination for the high antigen patients with LN‐FFR equal to 10%. The results are shown in Figure S6 (Supporting Information).

**Figure 7:**
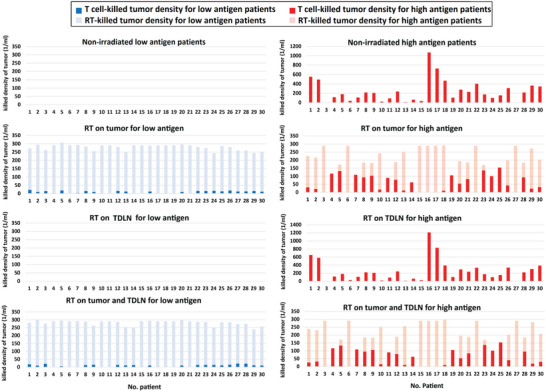
Comparison of the fractions of cancer cells killed by radiation therapy (“RT‐killed”) and by the immune response (“T‐cell killed”). Results for 30 independent simulations are shown for low (blue, left) and high (red, right) antigen producing cases. RT was applied to the tumor only, to the TDLN only, or to both tumor and TDLN.

To quantitatively investigate the importance of RT for treatment of low and high antigen patients, we quantified the contributions of the anti‐tumor immune response and the radiation‐induced cytotoxicity on tumor size reduction separately (**Figure** **7**). For high antigen patients the ratio of contributions to tumor killing due to RT and T cells have the same order of magnitude (Figure 7). However, for low antigen patients, the RT has a larger impact on tumor killing compared with the T cell mediated anti‐tumor immunity. In this case, RT is responsible for between 92 to 100% of the cell death, while the T cells are responsible for between 0 to 8% cancer cell killing (Figure 7). These results reinforce the result that low antigen patients may receive significant benefits from RT. However, high antigen producing tumors can be eradicated by immune response with or without RT. In some high antigen patients with delayed T cell activation, and thus higher T cell‐killed fraction (No. patient 15–17), RT of the tumor will reduce the antigen source and delay systematic circulation of antigen, which will stop T cell activation. Therefore, for high antigen patients, the application of radiation to the tumor may be counter‐productive.

## 3 Conclusion

Colocalization of naïve T cells with tumor antigen is a necessary but potentially inefficient step in anti‐tumor immunity. Before an immune response can be mounted against a tumor, T cells that recognize tumor antigen have to be activated and expanded. Although much research has focused on therapeutic modulation of immune checkpoint (IC) programs, relatively little is known about the systemic events the lead to T cell activation and their accumulation in the tumor. Here, we developed a model that simulates tumor growth, antigen production, the accumulation of antigen in lymph nodes and the trafficking of T cells, the IC mechanism for tumor‐immune interactions, and the implicit and explicit effects of radiotherapy on cancer clearance through the immunogenic and non‐immunogenic mechanisms. The simulations suggest that T cell activation depends strongly on two processes: the partitioning of T cells into lymph nodes as they circulate through the blood and the production rate of antigen by the tumor. Increasing the fraction of nT‐cells entering a lymph node significantly enhances the probability of activation in patients with high and medium antigen production rates and can considerably decrease the time required for activation in patients with high antigen production. Increasing LN‐FFR or antigen production can decrease the time needed for T cell activation.

The model has several limitations that should be considered as we interpret the results. For example, we use a simplified anatomy, with the multiple LNs associated with each organ “lumped” together into a representative node for that organ. We also have not included mechanisms of T cell retention in the LNs due to immune status or antigen level, which probably affect residence times. Furthermore, we did not explicitly consider T cell activation in the spleen, although this is likely justified, as blood‐borne antigen is highly diluted and accumulates in lower levels in the spleen compared with the draining lymph nodes. Note also that, in addition to the transport limitations studied here, defects in antigen processing and presentation machinery and the presence of immunosuppressive cell types can also determine the effectiveness of an anti‐tumor immune response. Other limitations are that the time course analyzed is limited, and the dynamics of T cell activation and tumor killing are necessarily simplified. Finally, we assume that immune checkpoint blockade is persistent and nearly complete, and the exhaustion rate of effector T cells is very small.^[^ ^45^, ^54^, ^55^ ^]^ These assumptions simplified the analysis, allowing us to focus on the systemic transport limitations rather than biological impediments to T cell activation and killing. Where significant T cell exhaustion occurs, a continued antitumor immune response would require the activation of additional nT‐cell clones as previous effector T cells become ineffective.^[^ ^41^, ^56^ ^]^ This process encompasses complex dynamics that are largely unknown, but that would be expected to amplify the importance of the stochastic processes explored here.

The simulations highlight the importance of tumor draining lymph nodes for the initiation of tumor immunity. Activation of nT‐cells in TDLNs can be compromised for various reasons, including inhibition by cytokines produced in the tumor and physical removal of the LN(s) during cancer surgery. Hence, we also examined the anti‐tumor immune response following TDLN removal (Figure S7, Supporting Information). Mathematical removal of the TDLN results in a delay in T cell activation because antigen accumulation in other lymph nodes is slower, and activation is consequently delayed compared with activation in the TDLN. Clinically, elective nodal irradiation, in which the tumor draining lymph nodes are targeted by the radiation field, is a common strategy designed to minimize regional recurrence. However, there is concern that the tumor draining lymph nodes should be spared from radiation therapy, as these are the sites of immune cell activation and expansion.^[^ ^57^, ^58^ ^]^ Consistent with this, our results suggest that nodal irradiation can be counter‐productive in patients with tumors that have low antigen production, because of toxic effects on proliferating T cells in the active nodes. This effect is less prominent when tumor antigen production is high because nT‐cell activation is more frequent, and not rate‐limiting. This agrees with clinical and experimental evidence showing that lymph node irradiation increases local and metastatic tumor growth, decreases the systemic immune response, and decreases antigen‐specific T cells and epitope spreading.^[^ ^57^ ^]^

In addition to concerns about LN irradiation, there are emerging questions about the appropriate use of therapies for killing cancer cells and enhancing antigen/neoantigen production in the context of immunotherapy. Specifically, it has been shown that radiation therapy applied to cancer cells can enhance tumor immunogenicity and creation of neoantigens, thus improving the response to ICB therapy.^[^ ^59^ ^]^ Our model supports this, predicting that irradiation of the tumor can enhance immunotherapy. However, it suggests that radiation should be considered carefully: radiation applied to the tumor in patients where the antigen level is already sufficient for successful ICB therapy may decrease the source of antigen, potentially abrogating the immune response in these tumors. The simulations also suggest that the timing of ICB therapy should be guided by the antigen production rate and the time window when T cell activation is most likely to occur.

## Conflict of Interest

L.L.M. is a consultant for SimBiosys and receives equity from Bayer.

## Supporting information

Supporting Information

## Data Availability

The data that support the findings of this study are available in the supplementary material of this article.

## References

[1] M. K. Callahan , J. D. Wolchok , Semin. Oncol. 2015, 42, 573. 26320062 10.1053/j.seminoncol.2015.05.008

[2] M. A. Postow , M. K. Callahan , J. D. Wolchok , J. Clin. Oncol. 2015, 33, 1974. 25605845 10.1200/JCO.2014.59.4358 PMC4980573

[3] K. Sanchez , D. Page , H. L. McArthur , Curr. Probl. Cancer 2016, 40, 151. 27855963 10.1016/j.currproblcancer.2016.09.009

[4] K. M. Mahoney , P. D. Rennert , G. J. Freeman , Nat. Rev. Drug Discovery 2015, 14, 561. 26228759 10.1038/nrd4591

[5] D. Fukumura , J. Kloepper , Z. Amoozgar , D. G. Duda , R. K. Jain , Nat. Rev. Clin. Oncol. 2018, 15, 325. 29508855 10.1038/nrclinonc.2018.29 PMC5921900

[6] T. D. Wu , S. Madireddi , P. E. de Almeida , R. Banchereau , Y. J. Chen , A. S. Chitre , E. Y. Chiang , H. Iftikhar , W. E. O′Gorman , A. Au‐Yeung , C. Takahashi , L. D. Goldstein , C. Poon , S. Keerthivasan , D. E. de Almeida Nagata , X. Du , H. M. Lee , K. L. Banta , S. Mariathasan , M. Das Thakur , M. A. Huseni , M. Ballinger , I. Estay , P. Caplazi , Z. Modrusan , L. Delamarre , I. Mellman , R. Bourgon , J. L. Grogan , Nature 2020, 579, 274. 32103181 10.1038/s41586-020-2056-8

[7] M. F. Fransen , M. Schoonderwoerd , P. Knopf , M. G. M. Camps , L. J. A. C. Hawinkels , M. Kneilling , T. Van Hall , F. Ossendorp , JCI Insight 2018, 3, 124507. 30518694 10.1172/jci.insight.124507 PMC6328025

[8] M. H. Spitzer , Y. Carmi , N. E. Reticker-Flynn , S. S. Kwek , D. Madhireddy , M. M. Martins , P. F. Gherardini , T. R. Prestwood , J. Chabon , S. C. Bendall , L. Fong , G. P. Nolan , E. G. Engleman , Cell 2017, 168, 487. 28111070 10.1016/j.cell.2016.12.022 PMC5312823

[9] G. Bogle , P. R. Dunbar , Immunol. Cell Biol. 2010, 88, 172. 19884904 10.1038/icb.2009.78

[10] D. Jones , E. R. Pereira , T. P. Padera , Front. Oncol. 2018, 8, 36. 29527513 10.3389/fonc.2018.00036 PMC5829610

[11] K. Murphy , C. Weaver , Janeway's Immunobiology, 9th ed., Garland Science, New York 2016.

[12] A. S. Perelson , F. W. Wiegel , J. Theor. Biol. 2009, 257, 9. 19084024 10.1016/j.jtbi.2008.11.007 PMC2878816

[13] G. Lythe , R. E. Callard , R. L. Hoare , C. Molina‐París , J. Theor. Biol. 2016, 389, 214. 26546971 10.1016/j.jtbi.2015.10.016 PMC4678146

[14] V. I. Zarnitsyna , B. D. Evavold , L. N. Schoettle , J. N. Blattman , R. Antia , Front. Immunol. 2013, 4, 485. 24421780 10.3389/fimmu.2013.00485 PMC3872652

[15] M. K. Jenkins , J. J. Moon , J. Immunol. 2012, 188, 4135. 22517866 10.4049/jimmunol.1102661 PMC3334329

[16] U. H. von Andrian , C. R. Mackay , N. Engl. J. Med. 2000, 343, 1020. 11018170 10.1056/NEJM200010053431407

[17] A. A. Davis , V. G. Patel , J. Immunother Cancer 2019, 7, 278. 31655605 10.1186/s40425-019-0768-9 PMC6815032

[18] T. Powles , L. Morrison , Nat. Rev. Urol. 2018, 15, 585. 30030491 10.1038/s41585-018-0056-3

[19] K. Aslan , V. Turco , J. Blobner , J. K. Sonner , A. R. Liuzzi , N. G. Nunez , D. de Feo , P. Kickingereder , M. Fischer , E. Green , A. Sadik , M. Friedrich , K. Sanghvi , M. Kilian , F. Cichon , L. Wolf , K. Jahne , A. von Landenberg , L. Bunse , F. Sahm , D. Schrimpf , J. Meyer , A. Alexander , G. Brugnara , R. Roth , K. Pfleiderer , B. Niesler , A. von Deimling , C. Opitz , M. O. Breckwoldt , et al., Nat. Commun. 2020, 11, 931. 32071302 10.1038/s41467-020-14642-0 PMC7028933

[20] I. X. Chen , K. Newcomer , K. E. Pauken , V. R. Juneja , K. Naxerova , M. W. Wu , M. Pinter , D. R. Sen , M. Singer , A. H. Sharpe , R. K. Jain , Proc. Natl. Acad. Sci. USA 2020, 117, 23684. 32907939 10.1073/pnas.2002806117 PMC7519254

[21] D. S. Park , M. Robertson‐Tessi , K. A. Luddy , P. K. Maini , M. B. Bonsall , R. A. Gatenby , A. R. A. Anderson , Cancer Res. 2019, 79, 5302. 31387920 10.1158/0008-5472.CAN-18-3712 PMC6801094

[22] K.‐A. Norton , C. Gong , S. Jamalian , A. Popel , Processes 2019, 7, 37. 30701168 10.3390/pr7010037 PMC6349239

[23] K. N. Margaris , R. A. Black , J. R. Soc. Interface 2012, 9, 601. 22237677 10.1098/rsif.2011.0751 PMC3284143

[24] G. E. Mahlbacher , K. C. Reihmer , H. B. Frieboes , J. Theor. Biol. 2019, 469, 47. 30836073 10.1016/j.jtbi.2019.03.002 PMC6579737

[25] J. D. Butner , D. Fuentes , B. Ozpolat , G. A. Calin , X. Zhou , J. Lowengrub , V. Cristini , Z. Wang , IEEE Trans. Biomed. Eng. 2020, 67, 1450. 31603768 10.1109/TBME.2019.2938485 PMC8445608

[26] N. S. Adrar , K. Madani , S. Adrar , PharmaNutrition 2019, 7, 100142.

[27] M. R. Nikmaneshi , R. K. Jain , L. L. Munn , PLoS Comput. Biol. 2023, 19, e1011131. 37289729 10.1371/journal.pcbi.1011131 PMC10249820

[28] M. R. Nikmaneshi , B. Firoozabadi , Biomech. Model. Mechanobiol 2022, 21, 1233. 35614373 10.1007/s10237-022-01587-0

[29] M. R. Nikmaneshi , B. Firoozabadi , A. Mozafari , Biotechnol. Bioeng. 2021, 118, 3871. 34133020 10.1002/bit.27863

[30] M. R. Nikmaneshi , B. Firoozabadi , A. Mozafari , L. L. Munn , Sci. Rep. 2020, 10, 3025. 32080250 10.1038/s41598-020-59658-0 PMC7033139

[31] C. K. Buhler , R. S. Terry , K. G. Link , F. R. Adler , Math. Biosci. Eng. 2021, 18, 6305. 34517535 10.3934/mbe.2021315 PMC10625481

[32] M. Jafarnejad , C. Gong , E. Gabrielson , I. H. Bartelink , P. Vicini , B. Wang , R. Narwal , L. Roskos , A. S. Popel , AAPS J. 2019, 21, 79. 31236847 10.1208/s12248-019-0350-x PMC6591205

[33] H. Wang , O. Milberg , I. H. Bartelink , P. Vicini , B. Wang , R. Narwal , L. Roskos , C. A. Santa‐Maria , A. S. Popel , R. Soc. Open Sci. 2019, 6, 190366.. 31218069 10.1098/rsos.190366 PMC6549962

[34] O. Milberg , C. Gong , M. Jafarnejad , I. H. Bartelink , B. Wang , P. Vicini , R. Narwal , L. Roskos , A. S. Popel , Sci. Rep. 2019, 9, 11286. 31375756 10.1038/s41598-019-47802-4 PMC6677731

[35] S. Jamalian , C. D. Bertram , W. J. Richardson , J. E. Moore , Am. J. Physiol. ‐ Heart C 2013, 305, H1709. 10.1152/ajpheart.00403.2013 PMC3882543 24124185

[36] S. Jamalian , M. J. Davis , D. C. Zawieja , J. E. Moore , PLoS One 2016, 11, e0148384. 26845031 10.1371/journal.pone.0148384 PMC4742072

[37] J. E. Moore , C. D. Bertram , Annu. Rev. Fluid Mech. 2018, 50, 459. 29713107 10.1146/annurev-fluid-122316-045259 PMC5922450

[38] C. D. Bertram , C. Macaskill , J. E. Moore, Jr. , J. Biomech. Eng. 2019, 141, 111006. 31074761 10.1115/1.4043724 PMC6808046

[39] C. Kunert , J. W. Baish , S. Liao , T. P. Padera , L. L. Munn , Proc. Natl. Acad. Sci. USA 2015, 112, 10938. 26283382 10.1073/pnas.1508330112 PMC4568261

[40] J. W. Baish , C. Kunert , T. P. Padera , L. L. Munn , PLoS Comput. Biol. 2016, 12, e1005231. 27935958 10.1371/journal.pcbi.1005231 PMC5147819

[41] K. E. Yost , A. T. Satpathy , D. K. Wells , Y. Qi , C. Wang , R. Kageyama , K. L. Mcnamara , J. M. Granja , K. Y. Sarin , R. A. Brown , R. K. Gupta , C. Curtis , S. L. Bucktrout , M. M. Davis , A. L. S. Chang , H. Y. Chang , Nat. Med. 2019, 25, 1251. 31359002 10.1038/s41591-019-0522-3 PMC6689255

[42] M. R. Nikmaneshi , R. K. Jain , L. L. Munn , PLoS Comput. Biol. 2023, 19, e1011131. 37289729 10.1371/journal.pcbi.1011131 PMC10249820

[43] M. G. Harisinghani , A. O'Shea , Atlas of lymph node anatomy, Springer, Berlin 2013.

[44] V. V. Ganusov , J. Auerbach , PLoS Comput. Biol. 2014, 10, e1003586. 24830705 10.1371/journal.pcbi.1003586 PMC4022467

[45] B. Breart , P. Bousso , Eur. J. Immunol. 2016, 46, 2730. 27730626 10.1002/eji.201646550

[46] J. B. Hay , B. B. Hobbs , J. Exp. Med. 1977, 145, 31. 830789 10.1084/jem.145.1.31 PMC2180596

[47] Q. Wang , Y. Fang , C. Li , T. L. Leong , M. Provencio , I.‐J. Oh , Z. Zhang , C. Su , Transl. Lung Cancer Res. 2023, 12, 312. 36895937 10.21037/tlcr-23-83 PMC9989803

[48] O. Bylicki , P. Tomasini , G. Radj , F. Guisier , I. Monnet , C. Ricordel , L. Bigay-Game , M. Geier , C. Chouaid , C. Daniel , Eur. J. Cancer 2023, 183, 38. 36801605 10.1016/j.ejca.2023.01.014

[49] L. A. Huppert , A. I. Daud , J. Clin. Oncol. 2021, 39, 2637. 34138634 10.1200/JCO.21.00943

[50] O. Hamid , C. Robert , A. Daud , M. S. Carlino , T. C. Mitchell , P. Hersey , J. Schachter , G. V. Long , F. S. Hodi , J. D. Wolchok , A. Arance , J. J. Grob , A. M. Joshua , J. S. Weber , L. Mortier , E. Jensen , S. J. Diede , B. H. Moreno , A. Ribas , Eur. J. Cancer 2021, 157, 391. 34571336 10.1016/j.ejca.2021.08.013 PMC9350885

[51] D. M. Lussier , E. Alspach , J. P. Ward , A. P. Miceli , D. Runci , J. M. White , C. Mpoy , C. D. Arthur , H. N. Kohlmiller , T. Jacks , M. N. Artyomov , B. E. Rogers , R. D. Schreiber , Proc. Natl. Acad. Sci. USA 2021, 118, e2102611118. 34099555 10.1073/pnas.2102611118 PMC8214694

[52] H. Menon , D. Chen , R. Ramapriyan , V. Verma , H. B. Barsoumian , T. R. Cushman , A. I. Younes , M. A. Cortez , J. J. Erasmus , P. De Groot , B. W. Carter , D. S. Hong , I. C. Glitza , R. Ferrarotto , M. Altan , A. Diab , S. G. Chun , J. V. Heymach , C. Tang , Q. N. Nguyen , J. W. Welsh , J. Immunother. Cancer 2019, 7, 1.30612589

[53] Z. S. Buchwald , T. H. Nasti , J. Lee , C. S. Eberhardt , A. Wieland , S. J. Im , D. Lawson , W. Curran , R. Ahmed , M. K. Khan , J. Immunother. Cancer 2020, 8, e000867. 33028691 10.1136/jitc-2020-000867 PMC7542667

[54] S. E. Henrickson , T. R. Mempel , I. B. Mazo , B. Liu , M. N. Artyomov , H. Zheng , A. Peixoto , M. P. Flynn , B. Senman , T. Junt , H. C. Wong , A. K. Chakraborty , U. H. Von Andrian , Nat. Immunol. 2008, 9, 282. 18204450 10.1038/ni1559 PMC2698867

[55] K.‐A. G. Buela , R. L. Hendricks , J. Immunol. 2015, 194, 379. 25422507 10.4049/jimmunol.1402326 PMC4272918

[56] K. E. Yost , H. Y. Chang , A. T. Satpathy , Science 2021, 372, 130. 33833111 10.1126/science.abd1329

[57] B. Neupert , N. Olimpo , K. Nguyen , D. Nguyen , M. Knitz , M. Hoen , Nat. Commun. 2022, 13, 7015. 36385142 10.1038/s41467-022-34676-w PMC9668826

[58] L. B. Darragh , J. Gadwa , T. T. Pham , B. Van Court , B. Neupert , N. A. Olimpo , K. Nguyen , D. Nguyen , M. W. Knitz , M. Hoen , S. Corbo , M. Joshi , Y. Zhuang , M. Amann , X. J. Wang , S. Dow , R. M. Kedl , V. Samedi , M. K. Boss , S. D. Karam , Nat. Commun. 2022, 13, 7015. 36385142 10.1038/s41467-022-34676-w PMC9668826

[59] J. T. Poleszczuk , K. A. Luddy , S. Prokopiou , M. Robertson‐Tessi , E. G. Moros , M. Fishman , J. Y. Djeu , S. E. Finkelstein , H. Enderling , Cancer Res. 2016, 76, 1009. 26833128 10.1158/0008-5472.CAN-15-1423

