# Supplementary material for: Transport Barriers Influence the Activation of Anti‐Tumor Immunity: A Systems Biology Analysis
Source: Adv Sci (Weinh). 2023 Nov 10;10(36):2304076. doi: 10.1002/advs.202304076 (PMC10754116; doi:10.1002/advs.202304076)
Supplement: Supplementary file 1 — Supporting Information [file ADVS-10-2304076-s001.pdf]

ADVANCED SCIENCE
Open Access

# Supporting Information

for *Adv. Sci.*, DOI 10.1002/advs.202304076

Transport Barriers Influence the Activation of Anti-Tumor Immunity: A Systems Biology Analysis

*Mohammad R. Nikmaneshi, James W. Baish, Hengbo Zhou, Timothy P. Padera and Lance L. Munn**

# Supplemental Material

Analysis of systemic transport barriers for the activation of anti-tumor immunity

Mohammad R. Nikmaneshi[1], Timothy P. Padera[1], James W. Baish[2], and
Lance Munn.[1,*]

[1] Department of Radiation Oncology, Massachusetts General Hospital and Harvard Medical School, Boston, MA 02114, USA
[2] Biomedical Engineering, Bucknell University, Lewisburg, PA 17837, USA
* munn@steele.mgh.harvard.edu

CONTENTS

***Supplementary Data and Figures*** ........ 3

**Estimation of lymph node visitation probabilities** ........ 3

**Determination of the time threshold for tumor elimination (for the definition of responders)** ........ 3

**Impact of LN-FFR on radiotherapy responses:** ........ 5

**Comparison between immune checkpoint blockade (ICB), radiation and their combination:** ........ 8

**Removal of the TDLN delays immune activation:** ........ 8

**Model Parameter definitions:** ........ 10

***Detailed description of the model*** ........ 11

***I: Circulation and stochastic activation of naive T cells (nT cells)*** ........ 11

***II: Proliferation and circulation of activated T cells (effector T cells); Tumor killing*** ........ 11

**Tumor compartment** ........ 12
- a) T-cell trafficking through the blood circulation and tumor tissue: ........ 12
- b) Tumor cell proliferation, angiogenesis and death: ........ 13
- c) Effect of radiation on non-cancerous cells: ........ 14
- d) Antigen distribution in the blood circulation and tumor interstitium: ........ 15
- e) T-cell trafficking to the tumor draining lymph node (TDLN) ........ 15
- f) Antigen distribution in TDLN ........ 16

**Lung** ........ 16
- a) T-cell trafficking in the blood and interstitial compartments ........ 16
- b) Antigen distribution in blood and tissues ........ 17
- c) T-cell trafficking in the LN of the lung ........ 17
- d) Antigen distribution in LNs of lung ........ 18

**Liver, Spleen and Intestine** ........ 18
- a) T-cell trafficking in blood vessels and interstitium of other organs, such as liver, intestine and spleen ........ 18
- b) antigen distribution in blood vessels and interstitium of liver, spleen, and intestine ........ 19
- c) T-cell trafficking in the abdominal LNs (LN_Abd) ........ 20
- d) Antigen distribution in LN of liver/spleen/intestines ........ 21

**Skin, Muscle, Bone, Brain and Kidney** ........ 22

a) T-cell trafficking in blood and interstitium of the *i*th organ, including skin, muscle, bone, brain and kidney: 22
b) Antigen distribution in blood vessels and interstitium 22
c) T-cell trafficking in the LN of the ith compartment including skin, muscle, bone, brain and kidney: 22
d) Antigen distribution in LN of compartment *i* 23

**Heart** 23
a) T-cell recirculation in arterial and venous blood flows 23
b) Arterial and venous recirculation of antigen 24
c) Mass conservation equations 25

**Model validation** 25

**Table S1. Model parameters** 26

***References*** 31

## Supplementary Data and Figures

### Estimation of lymph node visitation probabilities

Only a small fraction of total blood flow goes through lymph nodes, and each time a naive T cell makes a pass through the blood circulation, it can take a different path. Because there is no apparent mechanism for directing a blood-borne cell to one organ rather than another, or to the lymph node circulation rather than other parallel arterial paths, we assume these to be random processes. Based on this, and considering known circulation times and volumes, we can make a rough estimation of the probability that a specific naive T cell visits a lymph node that contains antigen (Figure 1S). In this estimation, we assume the volume of a human is 66 L and the number of organs (lymphosomes) is 8 (as in the simulations). The average volume of all nodes in each lymph node region (complex of multiple LNs region, each region approximately 63 LNs) is 34.7 ml. With this, the fractional volume of a single LN cluster relative to the body volume is $5.26\times10^{-4}$. Therefore, the fractional volume of each LN is equal to $5.26\times10^{-4}/63 = 8.34\times10^{-6}$.

In humans, blood circulates approximately once per minute. However, immune cells do not circulate this quickly because of significant residence times in capillary beds and lymph nodes. We assume that in the case that the nT cells does not enter a lymph node, the average circulation time is approximately 3 min due to the resistance in capillary beds [1]. If the nT cell enters a lymph node, the residence time is much longer (~10 hr) [1]. According to our stochastic model, each nT cell finds a LN region ~1.33 time per day (see figure 2 of the main text). If the residence time of a nT cell in a LN is 10hr, then on average, each nT cell is sequestered in a lymph node for 1.33×10 hours each day. Therefore, the effective average time that a nT cell actively circulates each day can be approximated by (24 hr-13.33 hr) ×60 ~ 640 min. If we assume that the circulation time is 3 min through organs other than lymph nodes, then the cell can circulate (640/3)+1(for residency in LNs) ~ 214 times per day. Therefore, the probabilities that a nT cell visits any specific lymph node in a given day can be calculated by

$(1-( 1-8.34\times10^{-6})^{214}) \times 100= 0.18$ %.

We can extend this analysis to estimate the probability of a given nT cell visiting any positive node in a 24 hour period as a function of the number of positive nodes (Fig. S1).

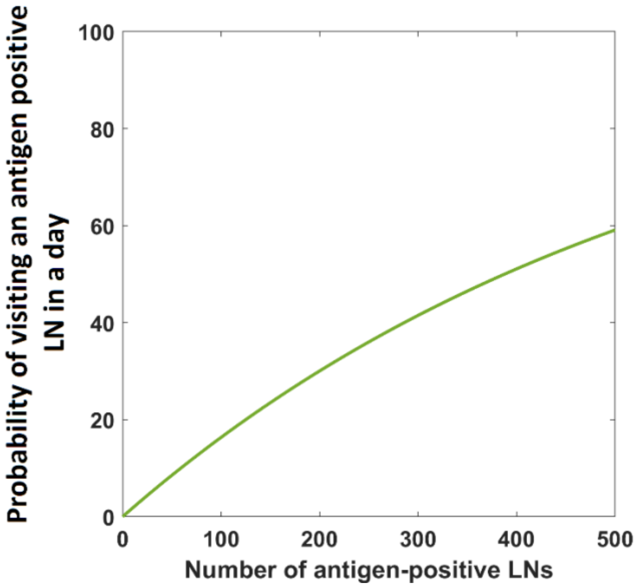


**Figure. S1.** Probability that a circulating n T cell visits an antigen-containing LN per day. Calculations are based on the relative blood flow through a lymph node in humans, estimated by tissue volumes. As more LNs receive antigen, the probability increases.

### Determination of the time threshold for tumor elimination (for the definition of responders)

We assume that the tumor has been growing for some time before the T cells are activated and begin to attack the cancer cells. In our simulations, even if a nT cell gets activated, if the T cells accumulate in the tumor too

slowly, then the tumor can out-proliferate the effector T cells and survive. Thus, there is a critical size of tumor above which it is too late for nT cell activation to result in tumor eradication. To estimate this threshold time for activation of T cells, we performed multiple runs of a simplified model. The simplified model has four parameters for vascular tumor growth, avascular tumor growth, T cell proliferation, and T cell killing of tumor cells. The tumor growth parameters of the simplified model were determined by curve fitting of the tumor growth profile for the complete model. The T cell proliferation rate and tumor -T cell reaction parameters were set by curve fitting with the tumor restrained profile of our complete model for the responder case with minimum activation time. With these assumptions, we can find the activation time that results in stable tumor size -- that is, where the T cells keep the tumor size stable. Later activation allows the tumor to continue growing (designated "non-responder") while earlier activation results in tumor eradication (designated "responder"). The analysis concludes that the critical time for activation is 4.62 days; activation at this time results in a tumor size reduction (TSR) ratio equal to ~ 0.7 at the 2 week time point (Fig. S2).

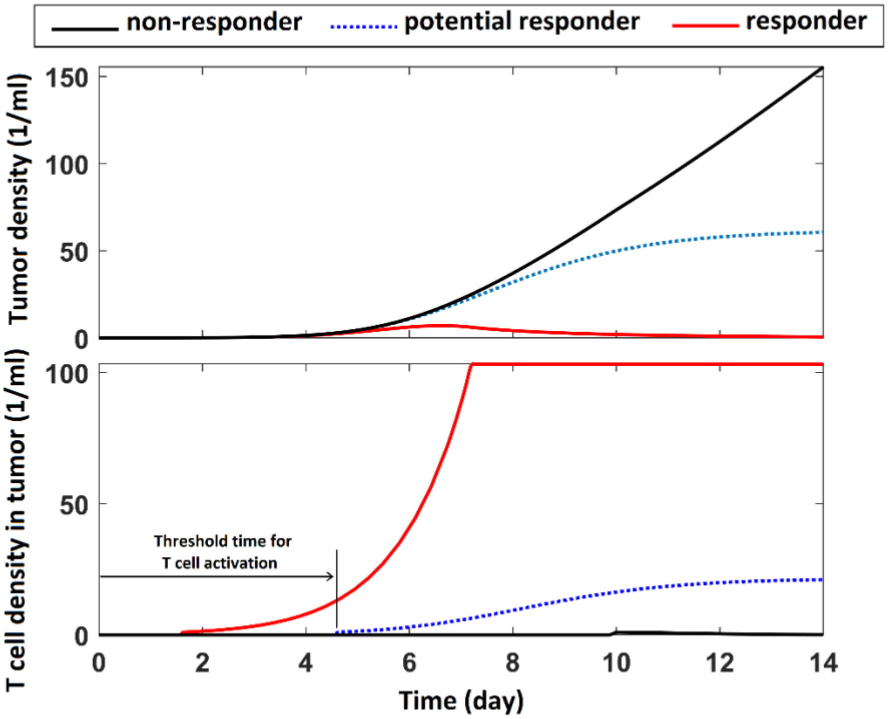


**Figure. S2.** Estimation of critical time for T cell activation to eliminate the tumor. The critical time is 4.62 days.

**Impact of LN-FFR on radiotherapy responses:**

To investigate the effect of LN-FFR on immune activation and radiation performance compared to the results of Figure 6 of the main text (where LN-FFR was fixed at 10%), we repeated the simulations with LN-FFR values of 5%, 1%, and 0.5%. The results of T cell accumulation in the TDLN and tumor growth for low and high antigen patients with LN-FFR equal to 5%, 1%, and 0.5% are shown in Figs. S3, S4 and S5, respectively.

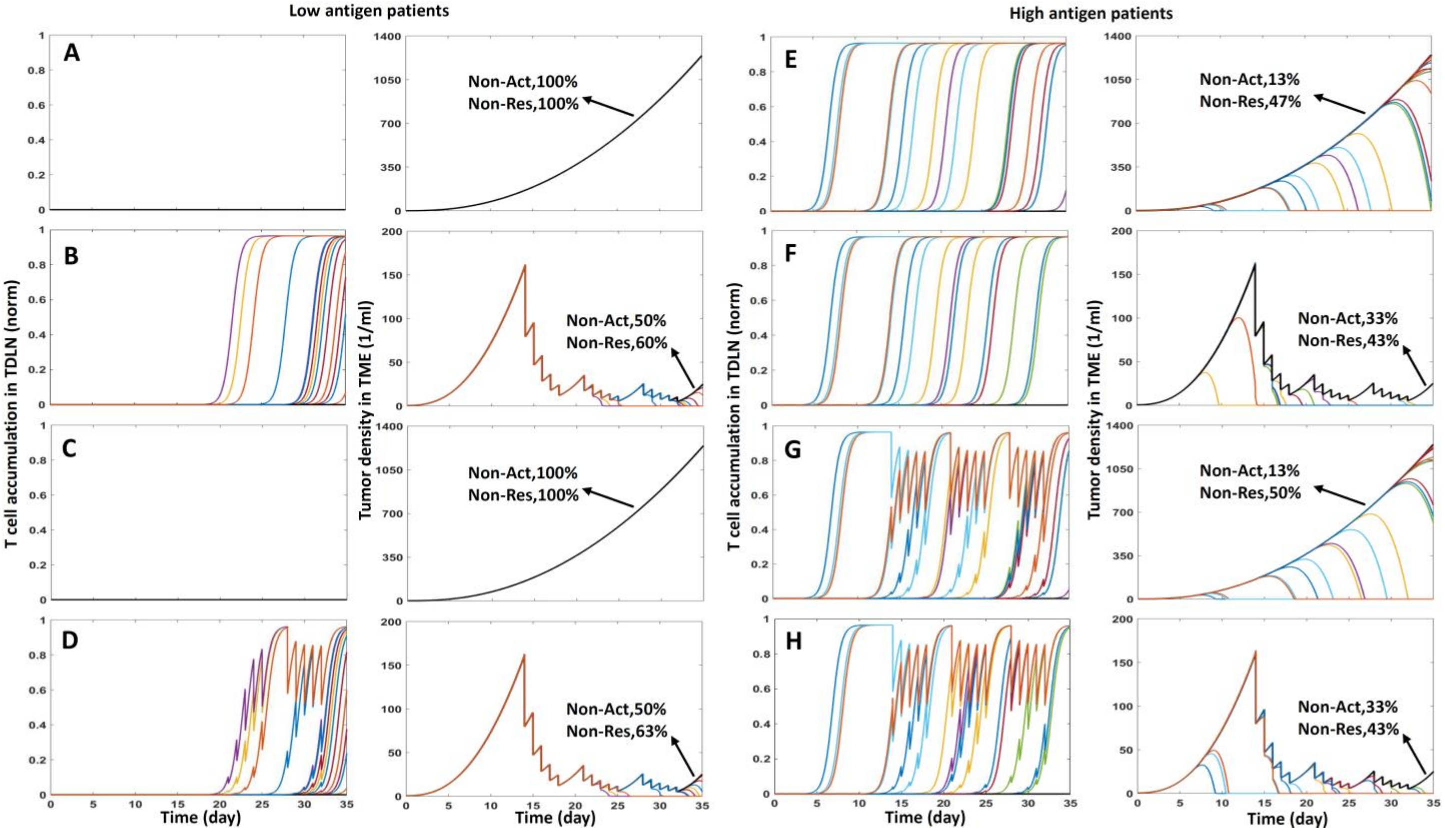


**Figure S3.** Effect of radiotherapy on tumor-immune response in low and high antigen patients with LN-FFR equal to 5%. 30 cases were simulated for each condition. RT starts on day 14, with daily dose of 2 Gy, except two days of weekend, administered over three weeks. A) Non-irradiated low antigen patients, B) RT on tumor of low antigen patients, C) radiation applied to the TDLN of low antigen patients, D) RT on tumor and TDLN of low antigen patients, E) non-irradiated high antigen patients, F) RT on tumor of high antigen patients, G) RT on TDLN of high antigen patients, H) RT on tumor and TDLN of high antigen patient. Black line shows patients in which T-cell activation does not occur. Colored lines show the T cell accumulation and tumor size in individual patients who have T-cell activation.

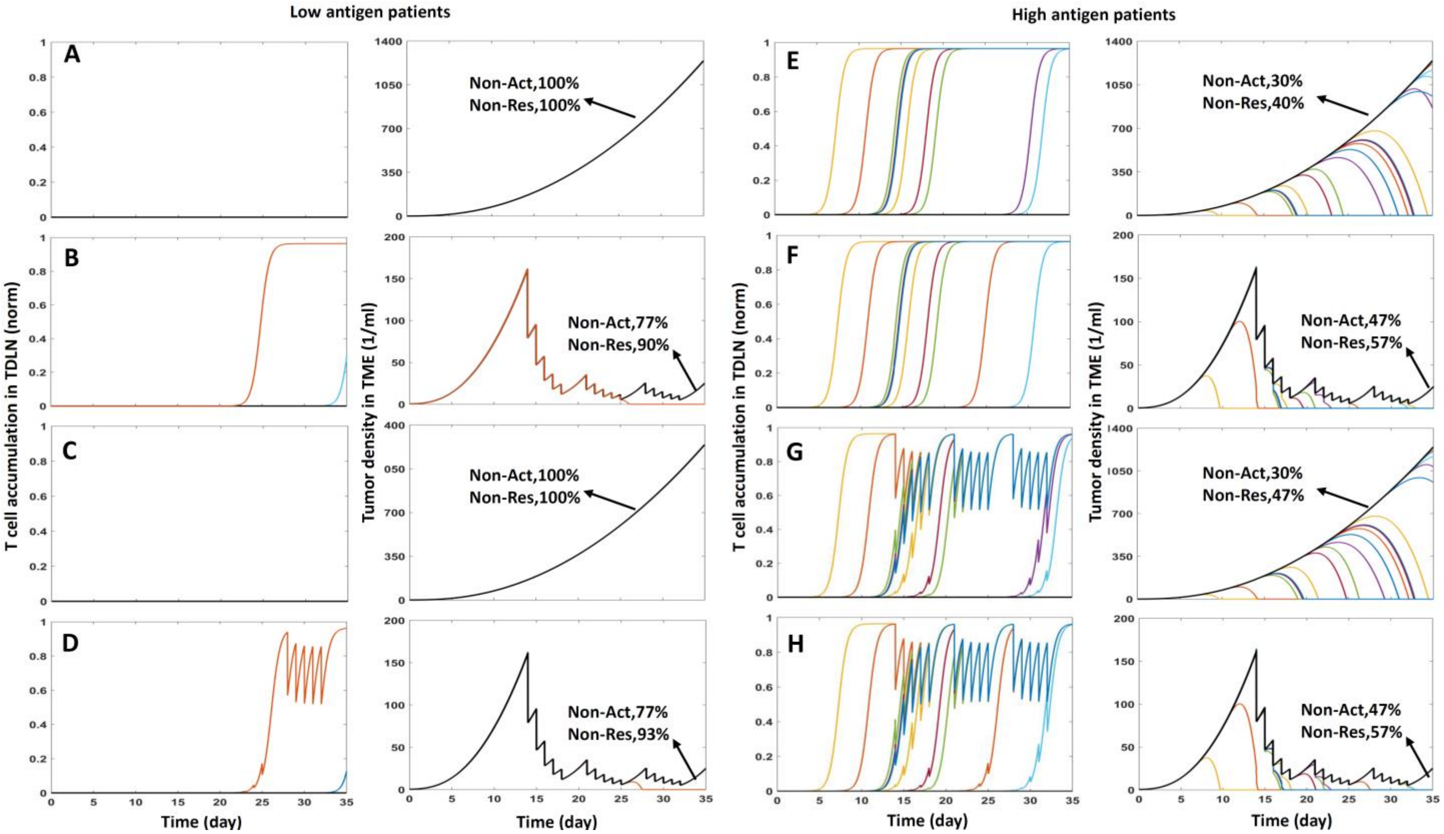


**Figure S4.** Effect of radiotherapy on tumor-immune response in low and high antigen patients with LN-FFR equal to 1%. 30 cases were simulated for each condition. RT starts on day 14, with daily dose of 2 Gy, except two days of weekend, administered over three weeks. A) Non-irradiated low antigen patients, B) RT on tumor of low antigen patients, C) radiation applied to the TDLN of low antigen patients, D) RT on tumor and TDLN of low antigen patients, E) non-irradiated high antigen patients, F) RT on tumor of high antigen patients, G) RT on TDLN of high antigen patients, H) RT on tumor and TDLN of high antigen patient. The black line shows patients in which T-cell activation does not occur. The colored lines show the TDLN T cell accumulation and tumor size in individual patients who have T-cell activation. Tumor size and T cell numbers are stochastically increased by activation, and are decreased by radiation treatment.

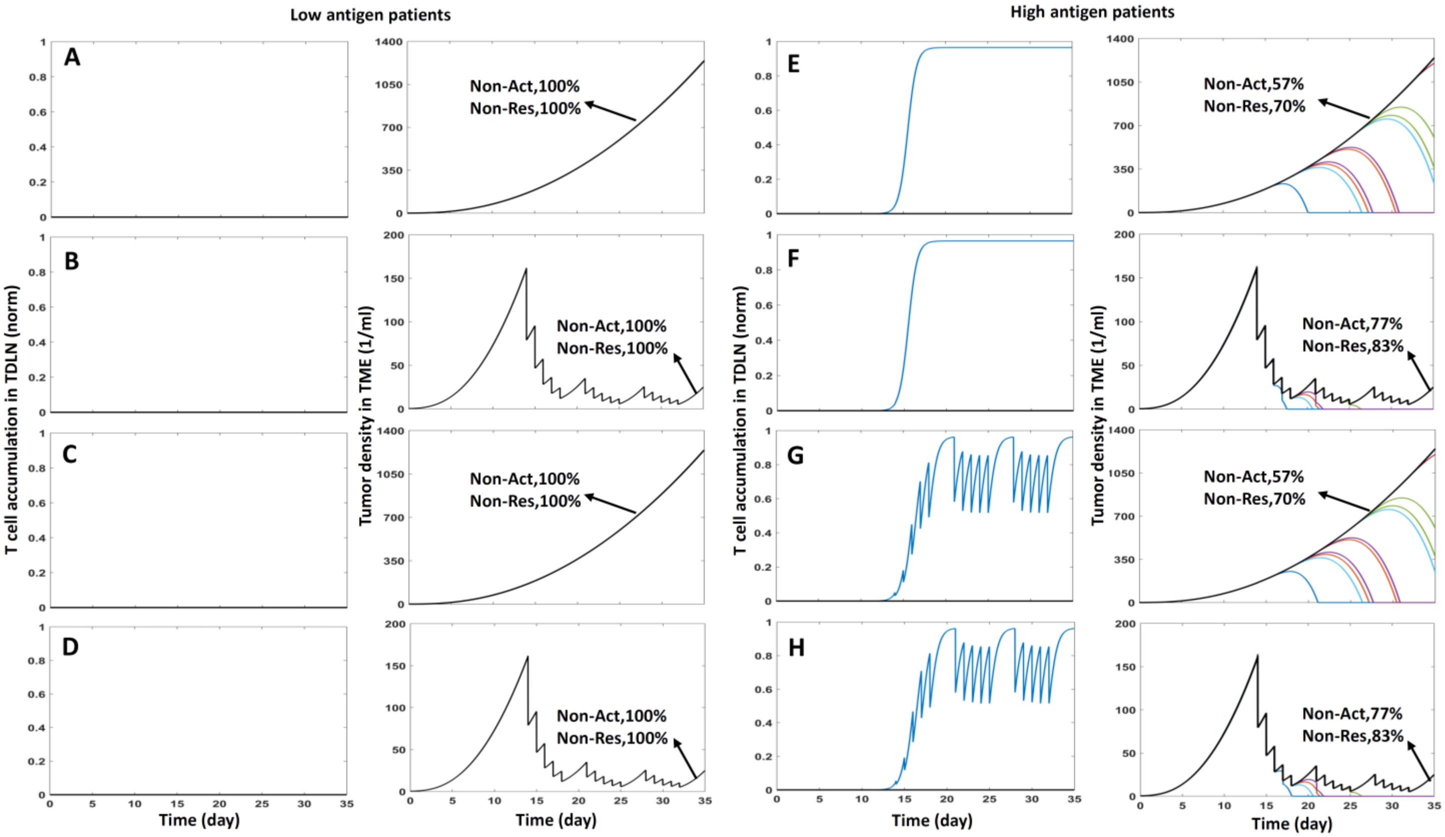


**Figure S5.** Effect of radiotherapy on tumor-immune response in low and high antigen patients with LN-FFR equal to 0.5%. 30 cases were simulated for each condition. RT starts on day 14, with daily dose of 2 Gy, except two days of weekend, administered over three weeks. A) Non-irradiated low antigen patients, B) RT on tumor of low antigen patients, C) radiation applied to the TDLN of low antigen patients, D) RT on tumor and TDLN of low antigen patients, E) non-irradiated high antigen patients, F) RT on tumor of high antigen patients, G) RT on TDLN of high antigen patients, H) RT on tumor and TDLN of high antigen patient. The black line shows patients in which T-cell activation does not occur. The colored lines show the TDLN T-cell accumulation and tumor size in individual patients who have T-cell activation. Tumor size and T cell numbers are stochastically increased by activation, and are decreased by radiation treatment.

Consistent with the results with 10% LN-FFR (Figure 6 of the main text), for high antigen patients, RT applied to the tumor decreases activation. RT applied to the TDLN affects tumor shrinkage (and calculated response rates) because of the damage to proliferating effector T cells in the node. Applying RT to both the tumor and TDLN combines these effects. For low antigen patients, radiation enhances activation, except in the 0.5% LN-FFR case, whether it is applied to the tumor or tumor and TDLN (see panels A-D of Figs. S3-S5). Decreasing the LN-FFR for high antigen patients consistently results in less activation and poorer response rates because the decreased LN-FFR delays activation in many patients. Similar to the results shown in Figure 6 of the main text, RT applied to the TDLN decreases response rates and has no effect on T cell activation. For low antigen patients with LN-FFR equal to 1% (Figure S4), RT applied to the tumor can cause activation for a few patients (Fig. S4, B and D). However, decreasing LN-FFR to 0.5% (Figure S5) results in no benefit for low antigen patients in terms of activation when RT is applied to the tumor. For high antigen patients, RT applied to the tumor delays T-cell activation (because less antigen source is available) and consequently decreases response rate. **Note that by decreasing LN-FFR there are some differences in the accumulation plots for T cell accumulation in the TDLN and the corresponding tumor growth curves (i.e., tumor shrinkage when there is no T cell activation in the TDLN). In these cases, T cell activation occurs in a non-tumor draining lymph node. For instance, the minimum LN-FFR, 0.5%, in Fig.S5 results in the most activation in other LNs such as lung LNs instead of TDLN (Fig. S5 E-H).**

## Comparison between immune checkpoint blockade (ICB), radiation and their combination:

We also compared RT, ICB and their combination for the patients with high antigen production and activated immune responses. Inhibition of T-cell killing results in only radiation-induced shrinking of the tumor (no ICB, Figure S6 A). Without radiation, but with T-cell killing, stochastic T-cell activation drives tumor shrinkage in some patients (Figure S6 B). Combining these effects result in additive tumor shrinkage, on a per-patient basis (Figure S6 C).

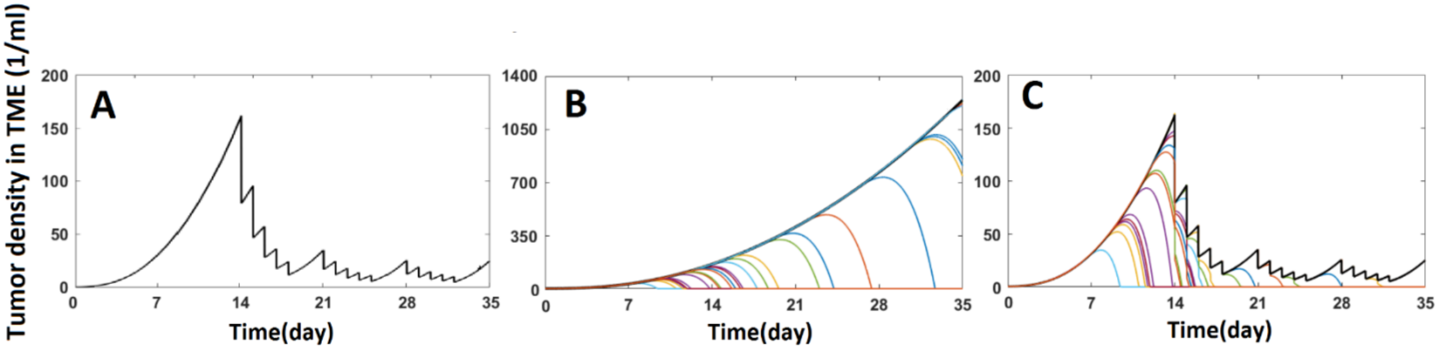


**Figure S6. Comparison of RT, ICB and their combination therapy for high antigen patients with LN-FFR equal to 10%**. A) RT applied to the tumor without the effect of ICB, so that activated T cells are unable to kill cancer cells. B) ICB is applied, allowing activated T cells to kill cancer cells. C) combination of ICB and RT applied to the tumor.

## Removal of the TDLN delays immune activation:

Reports in the literature have shown that the tumor draining lymph node (TDLN) is important for the initiation of tumor immunity, as it is a primary location of antigen accumulation [2]. To investigate this, we simulated removal of the TDLN and monitored activation rates. When the TDLN is removed, we assume that antigen can travel systemically via the thoracic duct and blood circulation to accumulate in other LNs. We further assume that blood-borne antigen partitions into LNs according to the LN-FFR. We simulated 100 cancer patients for conditions of high antigen production rate and an LN-FFR of 10% and plotted the time required for T cell activation (Fig. S7-A).

In these simulations, there was activation in the LNs associated with the lung and gastrointestinal tissues. We did not observe activation in other lymph nodes because of systemic dilution and low accumulation rates of antigen in other tissues. Antigen accumulated in the abdominal LN more quickly than the lung LN due to the higher flow volume of lymph to the central LNs, so the antigen concentration surpasses the threshold earlier in that LN (red and purple dashed lines, Fig. S7-A). The activation time for each patient is shown by the red and purple circles. Note that in the 40 day period, 72/100 patients had activation, while 28/100 did not. 62 patients had activation occur in the lung, while 9 had activation in the abdominal LN. Interestingly, $t_a$ is smaller for the abdominal LN (the antigen surpasses the threshold sooner), but $t_t$ is smaller in the lung (activation is more efficient in the lung, after antigen is present). This is because of the higher blood flow into the lungs vs. the GI tissue. This allows the nT cell to sample the lung LN more often than the GI LN.

The cases of early T cell activation in the abdominal LN resulted in larger tumor size reductions compared with activations in the lung LN (Fig. S7-B). Therefore, resecting the TDLN delays T cell activation, and consequently, leads to a significant delay in tumor killing by the T cells. Comparison between Fig. S7-B and Fig. 5 of the main text for high antigen patients with LN-FFR 10% shows that resecting the TDLN increases the initiation time of the response from one week to five weeks. Therefore, compared to patients with an intact, functional TDLN, a delay around four weeks is conceivable for administrating ICB treatment for patients without TDLN.

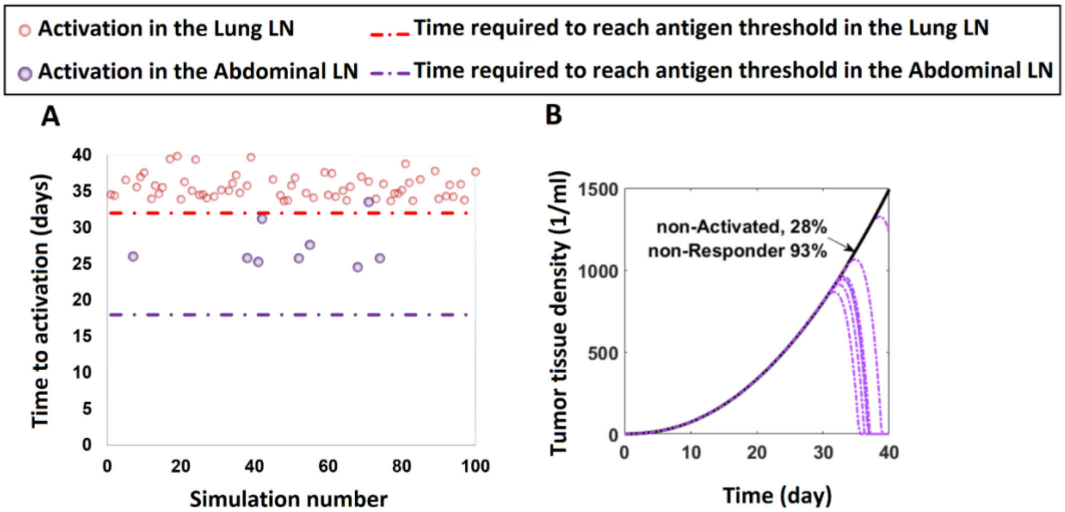


**Figure S7. Removal of the TDLN affects T cell activation and tumor size reduction.** A) After mathematical removal of the TDLN, we ran 100 simulations and recorded the time and location of nT cell activation. Activation time is indicated in pink for the lung LN and in violet for the central LN. B) Tumor size profiles of patients with TDLN resection over 40 days.

**Model Parameter definitions:**

In this model, each organ includes a tissue subcompartment and a LN subcompartment indexed with *i* and *LNi*, respectively. $T^b$ and $A^b$ are free T cell and antigen concentrations in blood stream, $T^c$ captured T cell concentration on blood vessel inner surface, $T^{ins}$ and $A^{ins}$ are interstitial T cell and antigen concentrations, $T^a$ and $A^a$ are T cell and antigen concentrations in the arterial flow of heart, $T^v$ and $A^v$ are T cell and antigen concentrations in the venous flow of heart, *a* and *b* attachment and detachment rates of free T cells, $J_t$ and $J_a$ are respectively the transmigration rate of captured T cells passing through blood vessel wall and the perfusion rate of antigen across the vessel wall, $\varepsilon$ is natural decay of antigen in interstitium.

In terms of fluid dynamics parameters, *Q* and *L* are blood flow rate and lymphatic flow rate, $V^b$ and $V^{ins}$ are the averaged volumes of blood vessels and interstitium, $fi_t$ and $fi_a$ are fractions of interstitial T cells and antigen that are drained by lymphatic vessels, respectively, which get into the local lymphatic flow. *f* and (1-*f*) are the fractions of blood flow rate to respectively supply the tissue part and lymph node part of each organ, *ff* is a fraction of lymphatic flow of each organ that is directly released into the systematic lymphatic flow; the remainder (1-*ff*) is the fraction of lymphatic flow from each organ that is released into the associated lymph node (Fig. 1).

For antigen-induced T cell proliferation, *S* is tumor-induced antigen function, *P* is antigen-induced T cell proliferation function, $\rho$ proliferation rate of T cells in LN, $A^{th}$ is the minimum antigen concentration to cause T cells proliferation, $T^{th1}$ is the minimum concentration of T cells in LN to start local proliferation, $T^{th2}$ is the maximum concentration of T cells that can be placed into each LN's paracortex, and when reached instantly stops T cell proliferation. $a_c$, $a_{c,I}$ and $a_{c,RT}$ are the antigen production rates of alive cancer cells, immune-induced apoptotic cancer cells, and apoptotic tumor cells by radiation.

In terms of anti-cancer immunity, $k_1$, $k_{-1}$, *and* $k_2$ are respectively the rates of tumor-T cell complex formation, tumor-T cell complex degradation, PD-L1/PD-1-based tumor cell killing, and PD-L1-induced T cell exhaustion, $\alpha_{T,C}$ the concentration of tumor-T cell complex, $C_a$ the concentration of live tumor cells, $T^{exh}{}_{tumor}$ concentration of PD-L1$^+$(Treg)-induced exhausted T cell in tumor tissue, $C_{ap,I}$ and $C_{ap,RT}$ are the concentrations of PD-L1/PD-1-based and radiation-induced apoptotic cancer cells.

For the radiotherapy part of model, *SF* is the survival fraction of irradiated cells, $\xi$ is scale factor for the radiosensitivity of cells, $\alpha$ and $\beta$ are cell type-specific radiosensitivity parameters for cells, and D is dose of radiation. $\xi$, $\alpha$ and $\beta$ are cell type-specific parameters.

For the tumor growth model, $g_v$ and $g_{av}$ are constant maximum rates of vascular and avascular tumor growth, $\lambda_{a0}$ is M-M constant of avascular tumor growth, $\beta_{ag}$ and $\omega_{ag}$ are, respectively, the maximum rate of angiogenesis and the maximum rate of vessel disruption, $\alpha_{vegf0}$ and $\theta_{vegf0}$ are the M-M constant for VEGF-induced angiogenesis and vessel disruption.

## Detailed description of the model

## I: Circulation and stochastic activation of naive T cells (nT cells)

First, we keep track of nT cell circulation, and their entry into various lymph nodes (Fig. S8). This is calculated based on relative blood flow rates into various organs and lymph nodes, with residence times assumed as in Table 1S. If an nT cell visits a lymph node where antigen concentration is above the threshold value, it transforms into an activated (effector) T cell, and we transition to the model in the next section.

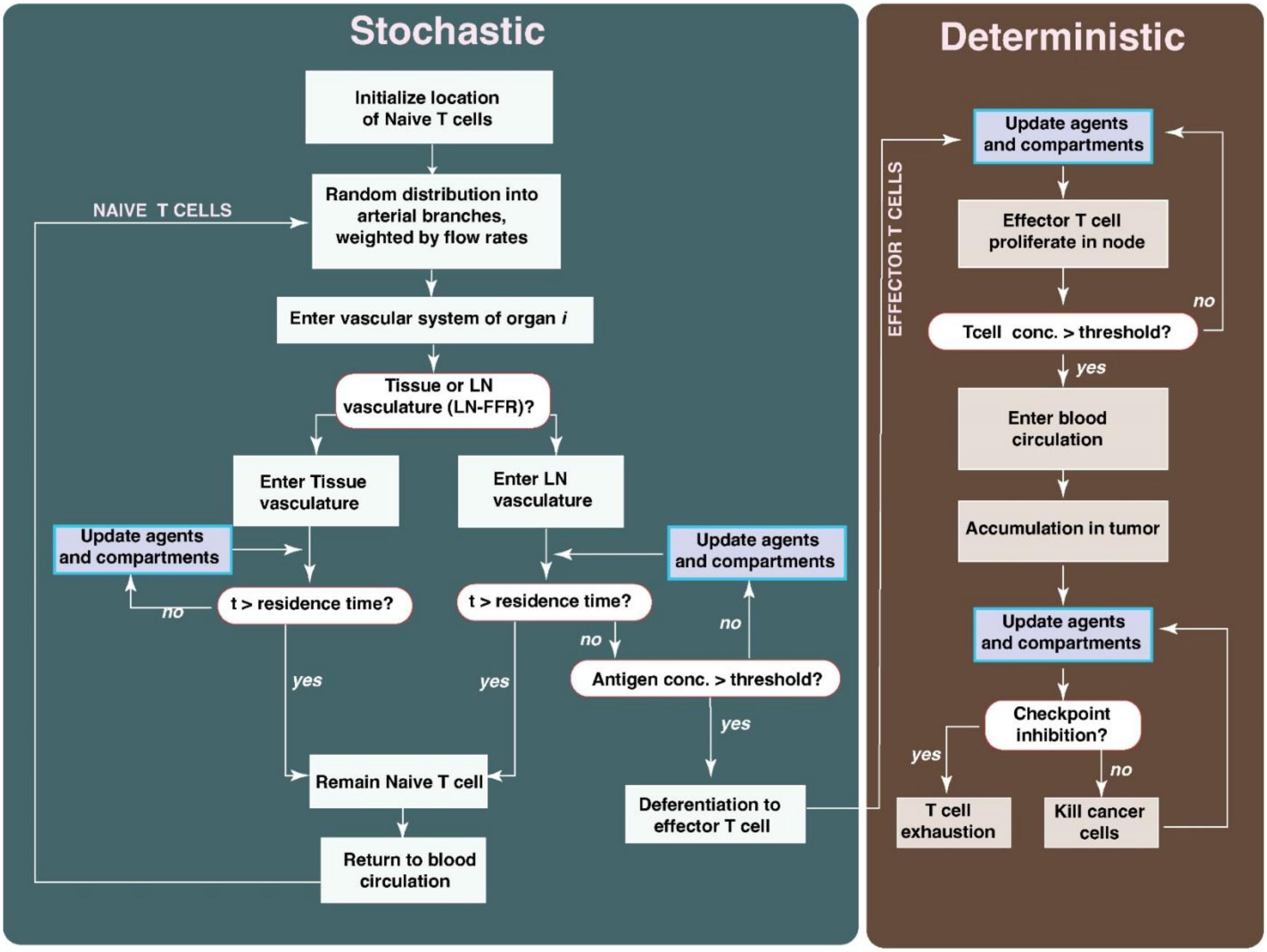


**Figure S8. computational Flow chart for the model.** The left hand side contains the stochastic algorithm for circulation and activation of naive T cells. The right hand side calculates the proliferation of activated T cells, their accumulation in the tumor and tumor killing. Note that for the presented simulations, the rate constant for checkpoint inhibition was set to 0.99, so there is nearly complete immune checkpoint inhibition and little T cell exhaustion. For the blue boxes "Update agents and compartments", we recalculate the tumor size, concentrations of antigen, T cells, angiogenic growth factors, tumor vasculature and flow rates in all compartments.

## II: Proliferation and circulation of activated T cells (effector T cells); Tumor killing

The rest of the model description for effector T cell distribution applies after a naive T cell finds an antigen-rich lymph node and becomes activated. After activation, we define three different states of the T cells: 1) free T cells circulating in the blood stream, 2) T cells attached to the blood vessel endothelium, and 3) T cells transmigrated into the tumor interstitium.

**Tumor compartment**

In the compartmental model, the tumor can be hosted by any of the organs. Here we simulate a tumor growing in the breast.

### a) T-cell trafficking through the blood circulation and tumor tissue:

The mass balance for T cells in the tumor bloodstream ($T^b_{tumor}$) is:

$$\frac{dT^b_{tumor}}{dt} = \begin{pmatrix} f_{tumor}\, Q_{tumor}\, T^b_{Heart} - (f_{tumor} Q_{tumor} - L_{tumor}) T^b_{tumor} \\ -a_{tumor}\, T^b_{tumor}\, V^b_{tumor} \\ +d_{tumor}\, T^c_{tumor}\, V^b_{tumor} \end{pmatrix} / V^b_{tumor} \quad (1a)$$

where the first term is the flow of T cells from the arterial blood into the tumor; the second term is the flow of T cells out of the tumor via the venous blood flow that equals inlet arterial flow minus filtered interstitial flow/lymphatic flow ($f\,Q$ - $L$); the third term represents the binding of T cells to the tumor endothelium, and the last term is the detachment of cells from the endothelium. In the tumor compartment, we assume that the vascular attachment and detachment rates ($a$ and $d$) of T cells depends on tumor size (Table. S).

The mass balance for the T cells attached to the vessel wall ($T^c_{tumor}$) is:

$$\frac{dT^c_{tumor}}{dt} = \left( a_{tumor}\, T^b_{tumor}\, V^b_{tumor} - d_{tumor}\, T^c_{tumor}\, V^b_{tumor} - J_{t,tumor}\, T^c_{tumor}\, V^b_{tumor} \right) / V^b_{tumor} \quad (1b)$$

Where the first term is the binding of T cells to the vessel wall, the second term is the detachment and the third term is transmigration.

The mass balance for T cells in the tumor tissue ($T^{ins}_{tumor}$) is:

$$\frac{dT^{ins}_{tumor}}{dt} = \begin{pmatrix} J_{t,tumor}\, T^c_{tumor}\, V^b_{tumor} - L_{tumor}\, T^{ins}_{tumor}\, fi_{t,tumor} \\ -\left( k_1 (1+\beta_v \frac{v}{v+\alpha_v})\, T^{ins}_{tumor}\, C_a - (k_{-1} + k_2\ p_r)\alpha_{T,C} \right) V^{ins}_{tumor} \end{pmatrix} / V^{ins}_{tumor} \quad (1c)$$

Where the first term is T cell transmigration into the tumor from blood vessels, the second term is T cell entry into lymphatic vessels; the third term represents the binding of T cells to cancer cells and the last term is de-conjugation of T cells from cancer cells, which can occur immediately after generation of tumor/T cell complex ($\alpha_{T,C}$) at a constant rate; $k_{-1}$, or after killing cancer cells by effector T cells (with the reaction rate equals $k_2\ p_r$ ).

We assume that effector T cells don't leave tumor interstitium, thus the drainage fraction of T cells in tumor tissue ($fi_{t,tumor}$) in the second term is set by zero (Table. S). Since the tumor tissue restricts T cell penetration into deep regions of tumor, we assume that angiogenic vessels can help T cells penetrate tumor tissue to increase the formation probability of cancer/T cell complex. Note that in these simulations, effector T cells are activated in lymph nodes and delivered to tissues via the blood stream and assumed to enter the tumor according to equation 1b. Therefore, we do not consider explicitly the effects of chemokines that might affect their migration once they arrive at the target site.

We assume that T cells become exhausted at a constant rate. The mass balance for exhausted T cells ($T^{exh}_{tumor}$) is:

$$\frac{dT_{tumor}^{exh}}{dt} = \left( k_2 \ (1-p_r) \ \alpha_{T,C} \right) \quad (1d)$$

Where $k_2 \ (1-p_r)$ is the rate of T cell exhaustion due to immune checkpoint (IC) mechanism of cancer cells. Note that for the present analysis, we assume that pr is ~1, so this mechanism is not significant.

**b) Tumor cell proliferation, angiogenesis and death:**

Tumor growth and angiogenesis: cancer cells proliferate, are bound by T cells and can de-conjugate and return to the active cell population.

The mass balance for active cancer cells, $C_a$ is:

$$\frac{dC_a}{dt} = \begin{pmatrix} g_v \left(\frac{v}{C_a+v}\right) C_a + g_{av} \left(\frac{C_a}{C_a+\lambda_{a0}}\right) + (k_{-1} + k_2 \ (1-p_r))\alpha_{T,C} \\ -k_1 \left(1+\beta_v \frac{v}{v+\alpha_v}\right) T_{tumor}^{ins} \ C_a \end{pmatrix} \quad (2a)$$

Where the first term is the vascularized tumor growth as a function of angiogenic vessels,$v$, and active cancer cells, $C_a$, the second term is avascularized tumor growth in the form of Michaelis–Menten model, the third term is the sumation of the de-conjugation rate of cancer cells from tumor/T cell complexes ($k_{-1} \ \alpha_{T,C}$) and the IC rate of cancer cells ($k_2 \ (1-p_r) \ \alpha_{T,C}$) (which is neglible here, since pr is ~0). The last term is the generation of tumor/T cell complexes with the help of angiogenic vessels.

The mass balance for cancer-immune cell conjugates $\alpha_{T,C}$ is:

$$\frac{d\,\alpha_{T,C}}{dt} = \left( k_1 \left(1+\beta_v \frac{v}{v+\alpha_v}\right) T_{tumor}^{ins} \ C_a - \left(k_{-1} + k_2\right)\alpha_{T,C} \right) \quad (2b)$$

Where the first term is production rate of complex formation as a function of angiogenic vessels that can help T cells penetrate deeper regions of cancer and make complex with inner cells, and the second term is reduction rate of cancer/T cell complex immediately after formation with reversibility rate ($k_{-1}$) and after doing reaction with T cells with rate $k_2$.

To model angiogenesis, we assumed that cancer cells secrete vascular endothelial growth factors (VEGF) that can stimulate endothelial cells to start angiogenesis. The concentration of tumor-induced VEGF, $c_{vegf}$**,** is given by:

$$\frac{dc_{vegf}}{dt} = \left( \eta_{ag} \ (C_a + \alpha_{T,C}) - \varepsilon_{vegf} \ c_{vegf} \right) \quad (2c)$$

where the first term is the production rate of VEGF by cancer cells (both de-conjugated and conjugated with T cells), and the second term is the natural decay of VEGF.

To model angiogenesis mechanism for vascular density, we developed equation (2d) based on VEGF concentration. Tumor-induced angiogenesis changes the angiogenic vessel density, $v$, through Eq. 2d:

$$\frac{dv}{dt} = \beta_{ag} \left( \frac{c_{vegf}}{c_{vegf} + \alpha_{vegf\,0}} \right) - \omega_{ag} \left( 1 - \frac{c_{vegf}}{c_{vegf} + \theta_{vegf\,0}} \right) v \quad (2d)$$

where the first term is the production of angiogenic vessels in response to VEGF and the second term is vascular pruning in response to low VEGF conditions.

Cancer cell death is induced by the immunogenic and non-immunogenic mechanisms. As the part of immunogenic mechanism of cancer cell death, the cancer cells are killed by effector T cells ($C_{ap,I}$) and the rate of this reaction is calculated by:

$$\frac{dC_{ap,I}}{dt} = \left( k_2 \;\; p_r \;\; \alpha_{T,C} \right) - \varepsilon_{ap,I} \;\; C_{ap,I} \qquad (2e)$$

In this model, non-immunogenic mechanisms of cancer cell death are caused by radiotherapy. The linear-quadratic model as a key tool in radiation biology provides a simple relationship between cell survival and delivered dose (set of Eqs. 2f) and has been used extensively to analyze and predict responses to ionizing radiation both in vitro and in vivo. To include the effect of radiation on cancer cell death, the linear-quadratic model of radiation damage given in Eq. 2f is applied for each application of RT [3].

Where $SF$ is the survival fraction of irradiated cells, $\xi$ is scale factor for the radiosensitivity of cells, $\alpha$ and $\beta$ are cell type-specific radiosensitivity parameters for cells, and D is dose of radiation.

$$C_a^{t^+} = C_a^{t^-} \; SF_c, \quad \alpha_{T,C}^{t^+} = \alpha_{T,C}^{t^-} \; SF_c \qquad (2f)$$

$$C_{ap,RT}^{t^+} = C_{ap,RT}^{t^-} + (C_a^{t^-} + \alpha_{T,C}^{t^-}) \; (1 - SF_c)$$

$$SF_c = \exp(-\xi_c(\alpha_c \; D + \beta_c \; D^2))$$

The decay of RT-killed cancer cells is represented by,

$$\frac{dC_{ap,RT}}{dt} = -\varepsilon_{ap,RT} \;\; C_{ap,RT} \qquad (2g)$$

**c) Effect of radiation on non-cancerous cells:**

The same mechanism can be applied for the vessels and T cells exposed to radiation therapy;

To determine the effect of radiation on angiogenic vessels, the linear-quadratic model of radiation is applied as Eq. 2h, after each application of RT,

$$v^{t^+} = v^{t^-} \; SF_v \qquad (2h)$$

$$v_{ap,RT}^{t^+} = v_{ap,RT}^{t^-} + v^{t^-} \; (1 - SF_v)$$

$$SF_v = \exp(-\xi_v(\alpha_v \; D + \beta_v \; D^2))$$

The decay of RT-killed angiogenic vessel can be represented by,

$$\frac{dv_{ap,RT}}{dt} = -\varepsilon_{ap,RT} \;\; v_{ap,RT}, \qquad (2i)$$

To determine the radiation effect on T cells the linear-quadratic model of radiation is applied as Eq. 2h, after each application of RT,

$$T_{tumor}^{\overset{+}{t}} = T_{tumor}^{\overset{-}{t}} \; SF_T \tag{2j}$$

$$T_{ap,RT}^{\overset{+}{t}} = T_{ap,RT}^{\overset{-}{t}} + T_{tumor}^{\overset{-}{t}} \; (1 - SF_T)$$

$$SF_T = \exp(-\xi_T (\alpha_T \; D + \beta_T \; D^2))$$

The decay of RT-killed T cells can be represented by,

$$\frac{dT_{ap,RT}}{dt} = -\varepsilon_{ap,RT} \; T_{ap,RT} \tag{2k}$$

**d) Antigen distribution in the blood circulation and tumor interstitium:**

Antigen is produced in the tumor and moves into the lymph and then the blood. The equation for antigen in the tumor blood supply ($A^b{}_{tumor}$) is:

$$\frac{dA_{tumor}^b}{dt} = \left( f_{tumor} \; Q_{tumor} \; A_{Heart}^b - \left( f_{tumor} \; Q_{tumor} - L_{tumor} \right) A_{tumor}^b - J_{a,tumor} \; A_{tumor}^b \; V_{tumor}^b \right) / V_{tumor}^b \tag{3a}$$

where the first term is the flow of antigen into the tumor via the bloodstream, the second term is antigen leaving the tumor via the blood and lymphatic systems and the last term is the filtration of antigen from the blood into the tumor tissue.

The source of antigen is live tumor cells and dying tumor cells (killed by radiation or T cells).

$$\frac{dA_{tumor}^{ins}}{dt} = \left( S \; V_{tumor}^{ins} + J_{a,tumor} \; A_{tumor}^b \; V_{tumor}^b - L_{tumor} \; A_{tumor}^{ins} \; fi_{a,tumor} - \varepsilon_{tumor} \; A_{tumor}^{ins} \; V_{tumor}^{ins} \right) / V_{tumor}^{ins} \tag{3b}$$

$$S = \left( a_c \, C_a + a_{cT} \, \alpha_{T,C} + a_{c,I} \, C_{ap,I} + a_{c,RT} \, C_{ap,RT} \right)$$

**e) T-cell trafficking to the tumor draining lymph node (TDLN)**

T cells enter the TDLN via the blood stream; they can exit with the blood flow, and the naïve T cell can be captured; however, effector T cells cannot be captured by attaching to the HEVs in the node:

$$\frac{dT_{TDLN}^b}{dt} = \begin{pmatrix} (1 - f_{tumor}) \; Q_{tumor} \; T_{tumor}^b \\ -\left( (1 - f_{tumor}) \; Q_{tumor} - L_{TDLN} \right) T_{TDLN}^b \end{pmatrix} / V_{TDLN}^b \tag{4a}$$

T cells enter from upstream tissue via lymph flow; they can also be killed by radiation treatments:

$$\frac{dT_{TDLN}^{ins}}{dt} = \begin{pmatrix} P(A_{TDLN}^{ins}, T_{TDLN}^{ins}) \\ +(1 - ff_{tumor}) \, L_{tumor} \; T_{tumor}^{ins} \; fi_{t,tumor} \\ -\left( (1 - ff_{tumor}) \, L_{tumor} + L_{TDLN} \right) \\ \times (H(T_{TDLN}^{th} - T_{TDLN}^{ins}) \; fi_{t,TDLN} + H(T_{TDLN}^{ins} - T_{TDLN}^{th})) T_{TDLN}^{ins} \\ \end{pmatrix} / V_{TDLN}^{ins} \tag{4b}$$

$$P(A_{TDLN}^{ins}, T_{TDLN}^{ins}) = \rho_{TDLN} \; T_{TDLN}^{ins} \; V_{TDLN}^{ins} \; H_{(A_{TDLN}^{ins} - A^{th})} \; H_{(T_{TDLN}^{ins} + nTcell)} \; H_{(T_{TDLN}^{th} - T_{TDLN}^{ins})}$$

where the first term is the proliferation rate of T cells in antigen-positive LNs, the second term is T cell flow from lymphatic flow of tissue into LNs, and the last term is the fraction of T cells that bypasses the LN and exits at the efferent lymphatic vessel. The proliferation rate of T cells is a function of antigen concentration in the LN, nT cell presence in the LN, and the level of effector T cells in the LN.

### f) Antigen distribution in TDLN

The mass balance for antigen in the blood circulation of the tumor draining lymph node ( $A^{b}_{TDLN}$ ) is:

$$\frac{dA^{b}_{TDLN}}{dt} = \begin{pmatrix} (1-f_{tumor})\, Q_{tumor}\, A^{b}_{heart} \\ -\left((1-f_{tumor})\, Q_{tumor} - L_{TDLN}\right) A^{b}_{TDLN} \\ -J_{a,TDLN}\, A^{b}_{TDLN}\, V^{b}_{TDLN} \end{pmatrix} / V^{b}_{TDLN} \tag{5a}$$

where the first term is the flow of antigen from the tumor to the LN in the blood; the second term is the flow of antigen in the efferent blood stream, and the last term is the antigen that crosses the blood vessel to enter the LN.

The mass balance for antigen within the tumor draining lymph node tissue ( $A^{ins}_{TDLN}$ ) is:

$$\frac{dA^{ins}_{TDLN}}{dt} = \begin{pmatrix} J_{a,TDLN}\, A^{b}_{TDLN}\, V^{b}_{TDLN} + (1-ff_{TDLN})\, L_{tumor}\, A^{ins}_{tumor}\, fi_{a,tumor} \\ -\left((1-ff_{TDLN})\, L_{tumor} + L_{TDLN}\right) A^{ins}_{TDLN}\, fi_{a,TDLN} \\ -\varepsilon_{TDLN}\, A^{ins}_{TDLN}\, V^{ins}_{TDLN} \end{pmatrix} / V^{ins}_{TDLN} \tag{5b}$$

where the first term is antigen entering the LN from the blood supply; the second term is the flow of antigen with the lymph via the afferent lymphatic vessel; the third term represents the fraction of antigen that bypasses the LN and exits at the efferent lymphatic vessel. The last term is the degradation of antigen in the node.

**Lung**

### a) T-cell trafficking in the blood and interstitial compartments

In other organs, such as lung, T cells enter and leave via the bloodstream; they also attach and detach from the vessel wall:

$$\frac{dT^{b}_{lung}}{dt} = \begin{pmatrix} Q_{lung}\, T^{v}_{Heart} - (Q_{lung} - L_{lung})\, T^{b}_{lung} \\ -a_{lung}\, T^{b}_{lung}\, V^{b}_{lung} \\ +d_{lung}\, T^{c}_{lung}\, V^{b}_{lung} \end{pmatrix} / V^{b}_{lung} \tag{6a}$$

The first term is the flow of T cells from the venous flow into the lung, the second term is the T cells leaving the lung via outlet of blood flow, the last terms are attachment and detachment rates of antigen from and to lung vessels.

T cells can extravasate into the interstitial compartment:

$$\frac{dT^{c}_{lung}}{dt} = \left( a_{lung}\, T^{b}_{lung}\, V^{b}_{lung} - d_{lung}\, T^{c}_{lung}\, V^{b}_{lung} - J_{t,lung}\, T^{c}_{lung}\, V^{b}_{lung} \right) / V^{b}_{lung} \tag{6b}$$

In this equation, the first term is the attachement rate of T cells to the lung vessels, the second term is the detachment rate of T cells from the lung vessels, and the last term is the transmigration rate of T cells from vessels to tissue.

T cells enter the lung tissue by transmigration, and leave via the lymphatic system:

$$\frac{dT_{lung}^{ins}}{dt}=\left(J_{t,lung}\,T_{lung}^{c}\,V_{lung}^{b}-L_{lung}\,T_{lung}^{ins}\,fi_{t,lung}\right)/V_{lung}^{ins} \tag{6c}$$

Where, the first term is the transmigration rate of T cells, and the second term is the T cell flow out of tissue part of lung.

## b) Antigen distribution in blood and tissues

Blood-borne antigen can enter other tissues, including lung. The mass balance for antigen concentration in the blood compartment of the lung is:

$$\frac{dA_{lung}^{b}}{dt}=\left(Q_{lung}\,A_{Heart}^{v}-\left(Q_{lung}-L_{lung}\right)A_{lung}^{b}-J_{a,lung}\,A_{lung}^{b}\,V_{lung}^{b}\right)/V_{lung}^{b} \tag{7a}$$

where the first term is the flow of antigen from the venous flow into the lung, the second term is the flow of antigen out of the lung, and the last term is trasnmigration;

Antigen leaves the tissue via lymphatic drainage, and can also degrade:

$$\frac{dA_{lung}^{ins}}{dt}=\begin{pmatrix}J_{a,lung}\,A_{lung}^{b}\,V_{lung}^{b}-L_{lung}\,A_{lung}^{ins}\,fi_{a,lung}\\ -\varepsilon_{lung}\,A_{lung}^{ins}\,V_{lung}^{ins}\end{pmatrix}/V_{lung}^{ins} \tag{7b}$$

where the first term is the trasnmigration of antigen into lung tissue from blood vessels, the second term is antigen leaving the lung interstitium via the lymphatic system and the last term is the natural decay of antigen.

## c) T-cell trafficking in the LN of the lung

T cells can enter/leave a LN via blood vessels; they attach and detach from HEVs:

$$\frac{dT_{LNlung}^{b}}{dt}=\begin{pmatrix}(1-f_{lung})(Q_{lung}-L_{lung})T_{lung}^{b}\\ -\left((1-f_{lung})(Q_{lung}-L_{lung})-L_{LNlung}\right)T_{LNlung}^{b}\end{pmatrix}/V_{LNlung}^{b} \tag{8a}$$

Where the first term is T cell flow from the outlet blood flow of the lung to the lung LNs and the second term is T cell flow leaving the LNs of lung via blood and lymphatic system.

T cells enter the node via transmigration or lymph flow; they can activate or flow out via lymph:

$$\frac{dT_{LNlung}^{ins}}{dt}=\begin{pmatrix}P(A_{LNlung}^{ins},T_{LNlung}^{ins})\\ +(1-ff_{lung})\,L_{lung}\,T_{lung}^{ins}\,fi_{t,lung}\\ -\left((1-ff_{lung})\,L_{lung}+L_{LNlung}\right)\\ \times(H(T_{LNlung}^{th}-T_{LNlung}^{ins})\,fi_{t,LNtumor}+H(T_{LNlung}^{ins}-T_{LNlung}^{th}))T_{LNlung}^{ins}\end{pmatrix}/V_{LNlung}^{ins} \tag{8c}$$

$$P(A_{LNlung}^{ins},T_{LNlung}^{ins})=\rho_{LNlung}\,T_{LNlung}^{ins}\,V_{LNlung}^{ins}\,H_{(A_{LNlung}^{ins}-A^{th})}\,H_{(T_{LNlung}^{ins}+nTcell)}\,H_{(T_{LNlung}^{th}-T_{LNlung}^{ins})}$$

where the first term is the proliferation rate of T cells in antigen-positive LNs of lung, the second term is T cell flow from lymphatic flow of tissue into LNs, and the last term is the fraction of T cells that bypasses the LN and exits at the efferent lymphatic vessel. The proliferation rate of T cells is a function of antigen concentration in the LN, n T cell presence in the LN, and the level of effector T cells in the LN.

**d) Antigen distribution in LNs of lung**

Antigen enters/leaves the LN blood supply via blood flow (and can extravasate):

$$\frac{dA^{b}_{LNlung}}{dt}=\begin{pmatrix}(1-f_{lung})(Q_{lung}-L_{lung})\;A^{b}_{lung}\\ -\left((1-f_{lung})(Q_{lung}-L_{lung})-L_{LNlung}\right)A^{b}_{LNlung}\\ -J_{a,LNlung}\;A^{b}_{LNlung}\;V^{b}_{LNlung}\end{pmatrix}/V^{b}_{LNlung} \quad (9a)$$

Antigen enters the LN via lymph flow; it can leave the LN via lymph flow, and degrade:

$$\frac{dA^{ins}_{LNlung}}{dt}=\begin{pmatrix}J_{a,LNlung}\;A^{b}_{LNlung}\;V^{b}_{LNlung}+(1-ff_{lung})\;\;L_{lung}\;A^{ins}_{lung}\;\;fi_{a,lung}\\ -\left((1-ff_{lung})\;\;L_{lung}+L_{LNlung}\right)A^{ins}_{LNlung}\;\;fi_{a,LNlung}\\ -\varepsilon_{LNlung}\;A^{ins}_{LNlung}\;\;V^{ins}_{LNlung}\end{pmatrix}/V^{ins}_{LNlung} \quad (9b)$$

## Liver, Spleen and Intestine

**a) T-cell trafficking in blood vessels and interstitium of other organs, such as liver, intestine and spleen.**

These organs have a connected network of LNs. We assume a common LN region for the abdominal LN to show this connectivity. The upstream blood flow can enter intestine, spleen and liver and after plasma filtration, the interstitial plasma flows of these three organs can collect into their common LN region.

For the liver

$$\frac{dT^{b}_{liver}}{dt}=\begin{pmatrix}f_{liver}\,(Q_{\text{int}estine}-L_{\text{int}estine})T^{b}_{\text{int}estine}+f_{liver}\,(Q_{spleen}-L_{spleen})T^{b}_{spleen}\\ +f_{liver}\,(Q_{liver}-Q_{\text{int}estine}-Q_{spleen}+L_{\text{int}estine}+L_{spleen})T^{a}_{Heart}\\ -(f_{liver}\;\;Q_{liver}-L_{liver})T^{b}_{liver}\\ -a_{liver}\,T^{b}_{liver}\,V^{b}_{liver}\\ +d_{liver}\,T^{c}_{liver}\,V^{b}_{liver}\end{pmatrix}/V^{b}_{liver} \quad (10a)$$

where the first and second terms are the flow of T cells from the outlet blood flows of intestine and spleen into the liver, the third term is the flow of T cells from the arterial blood into the liver; the fourth term is the flow of T cells out of the liver; the two last terms represent the binding of T cells to the liver endothelium and the detachment of T cells from the endothelium. In Eqs. 10b and 10c, the terms with the parameter *J* are transmigration of T cells into the liver interstitium.

$$\frac{dT^{c}_{liver}}{dt}=\left(a_{liver}\,T^{b}_{liver}\,V^{b}_{liver}-d_{liver}\,T^{c}_{liver}\,V^{b}_{liver}-J_{t,liver}\,T^{c}_{liver}\,V^{b}_{liver}\right)/V^{b}_{liver} \quad (10b)$$

$$\frac{dT_{liver}^{ins}}{dt}=\left( J_{t,liver}\, T_{liver}^{c}\, V_{liver}^{b} - L_{liver}\, T_{liver}^{ins}\, fi_{t,liver} \right)/ V_{liver}^{ins} \tag{10c}$$

For the spleen

$$\frac{dT_{spleen}^{b}}{dt}=\begin{pmatrix} Q_{spleen}\, T_{Heart}^{v} - (Q_{spleen} - L_{spleen}) T_{spleen}^{b} \\ -a_{spleen}\, T_{spleen}^{b}\, V_{spleen}^{b} \\ +d_{spleen}\, T_{spleen}^{c}\, V_{spleen}^{b} \end{pmatrix} / V_{spleen}^{b} \tag{10d}$$

where the first term is the flow of T cells from the arterial blood into the spleen; the second term is the flow of T cells out of the spleen; the third term represents the binding of T cells to the spleen endothelium, and the last term is the detachment of cells from the endothelium. In Eqs. 10e and 10f, the terms with *J* are transmigration of T cells into the spleen interstitium. The second term in Eq.10f is the drainage of T cell by filtration from the spleen interstitium to lymphatic vessels.

$$\frac{dT_{spleen}^{c}}{dt}=\left( a_{spleen}\, T_{spleen}^{b}\, V_{spleen}^{b} - d_{spleen}\, T_{spleen}^{c}\, V_{spleen}^{b} - J_{t,spleen}\, T_{spleen}^{c}\, V_{spleen}^{b} \right)/ V_{spleen}^{b} \tag{10e}$$

$$\frac{dT_{spleen}^{ins}}{dt}=\left( J_{t,spleen}\, T_{spleen}^{c}\, V_{spleen}^{b} - L_{spleen}\, T_{spleen}^{ins}\, fi_{t,spleen} \right)/ V_{liver}^{ins} \tag{10f}$$

For the intestine

$$\frac{dT_{\text{int}estine}^{b}}{dt}=\begin{pmatrix} Q_{\text{int}estine}\, T_{Heart}^{v} - (Q_{\text{int}estine} - L_{\text{int}estine}) T_{\text{int}estine}^{b} \\ -a_{\text{int}estine}\, T_{\text{int}estine}^{b}\, V_{\text{int}estine}^{b} \\ +d_{\text{int}estine}\, T_{\text{int}estine}^{c}\, V_{\text{int}estine}^{b} \end{pmatrix} / V_{\text{int}estine}^{b} \tag{10g}$$

where the first term is the flow of T cells from the arterial blood into the intestine; the second term is the flow of T cells out of the intestine; the third term represents the binding of T cells to the intestine endothelium, and the last term is the detachment of T cells from the intestine endothelium. In Eqs. 10h and 10l, the terms with *J* are transmigration of T cells into the intestine interstitium. The second term in Eq.10h is the drainage of T cell by filtration from the intestine interstitium to lymphatic vessels.

$$\frac{dT_{\text{int}estine}^{c}}{dt}=\begin{pmatrix} a_{\text{int}estine}\, T_{\text{int}estine}^{b}\, V_{\text{int}estine}^{b} - d_{\text{int}estine}\, T_{\text{int}estine}^{c}\, V_{\text{int}estine}^{b} \\ -J_{t,\text{int}estine}\, T_{\text{int}estine}^{c}\, V_{\text{int}estine}^{b} \end{pmatrix} / V_{\text{int}estine}^{b} \tag{10h}$$

$$\frac{dT_{\text{int}estine}^{ins}}{dt}=\left( J_{t,\text{int}estine}\, T_{\text{int}estine}^{c}\, V_{\text{int}estine}^{b} - L_{\text{int}estine}\, T_{\text{int}estine}^{ins}\, fi_{t,\text{int}estine} \right)/ V_{\text{int}estine}^{ins} \tag{10l}$$

**b) antigen distribution in blood vessels and interstitium of liver, spleen, and intestine**

For the liver

$$\frac{dA^b_{liver}}{dt}=\begin{pmatrix} f_{liver}\ (Q_{\text{int}estine}-L_{\text{int}estine})A^b_{\text{int}estine}+f_{liver}\ (Q_{spleen}-L_{spleen})A^b_{spleen} \\ +f_{liver}\ (Q_{liver}-Q_{\text{int}estine}-Q_{spleen}+L_{\text{int}estine}+L_{spleen})A^a_{Heart} \\ -\left(f_{liver}\ \ Q_{liver}-L_{liver}\right)A^b_{liver}-J_{a,liver}\ A^b_{liver}V^b_{liver} \end{pmatrix}/V^b_{liver} \quad (11a)$$

where the first and second terms are the flow of antigen into the liver via the bloodstream passing from intestine and spleen, the third term is the antigen flow from the arterial flow of heart into liver, the fourth term is antigen leaving the liver via the outlet blood flow and the last term is the filtration of antigen from the blood into the tumor tissue. In Eqs. 11b-f, the terms with J represent transmigration, the terms with $L$ and $Q$-$L$ are the antigen flows out via lymphatic system and the outlet blood flow. The ε terms are natural decay of antigen.

$$\frac{dA^{ins}_{liver}}{dt}=\begin{pmatrix} J_{a,liver}\ A^b_{liver}V^b_{liver}-L_{liver}\ A^{ins}_{liver}\ f_{a,liver} \\ -\varepsilon_{liver}\ A^{ins}_{liver}\ V^{ins}_{liver} \end{pmatrix}/V^{ins}_{liver} \quad (11b)$$

For the spleen

$$\frac{dA^b_{spleen}}{dt}=\begin{pmatrix} Q_{spleen}\ A^a_{Heart}-(Q_{spleen}-L_{spleen})A^b_{spleen} \\ -J_{a,spleen}\ A^b_{spleen}V^b_{spleen} \end{pmatrix}/V^b_{spleen} \quad (11c)$$

$$\frac{dA^{ins}_{spleen}}{dt}=\begin{pmatrix} J_{a,spleen}\ A^b_{spleen}V^b_{spleen}-L_{spleen}\ A^{ins}_{spleen}\ f_{a,spleen} \\ -\varepsilon_{spleen}\ A^{ins}_{spleen}\ V^{ins}_{spleen} \end{pmatrix}/V^{ins}_{spleen} \quad (11d)$$

For the intestines

$$\frac{dA^b_{\text{int}estine}}{dt}=\begin{pmatrix} Q_{\text{int}estine}\ A^a_{Heart}-(Q_{\text{int}estine}-L_{\text{int}estine})A^b_{\text{int}estine} \\ -J_{a,\text{int}estine}\ A^b_{\text{int}estine}V^b_{\text{int}estine} \end{pmatrix}/V^b_{\text{int}estine} \quad (11e)$$

$$\frac{dA^{ins}_{\text{int}estine}}{dt}=\begin{pmatrix} J_{a,\text{int}estine}\ A^b_{\text{int}estine}V^b_{\text{int}estine}-L_{\text{int}estine}\ A^{ins}_{\text{int}estine}\ fi_{a,\text{int}estine} \\ -\varepsilon_{\text{int}estine}\ A^{ins}_{\text{int}estine}\ V^{ins}_{\text{int}estine} \end{pmatrix}/V^{ins}_{\text{int}estine} \quad (11f)$$

## c) T-cell trafficking in the abdominal LNs (LN_Abd)

$$\frac{dT^b_{LN\_Abd}}{dt}=\begin{pmatrix} (1-f_{liver})(Q_{\text{int}estine}-L_{\text{int}estine})T^b_{\text{int}estine} \\ +(1-f_{liver})(Q_{spleen}-L_{spleen})T^b_{spleen} \\ +(1-f_{liver})\ (Q_{liver}-Q_{\text{int}estine}-Q_{spleen}+L_{\text{int}estine}+L_{spleen})T^a_{Heart} \\ -\left((1-f_{liver})\ Q_{liver}-L_{LN\_Abd}\right)T^b_{LN\_Abd} \\ \end{pmatrix}/V^b_{LN\_Abd} \quad (12a)$$

where the first term is the T cell flow in LNs via blood flow of the intestine, the second term is the T cell flow in LNs via blood flow of the spleen, the third term is the T cell flow from outflow of blood into the liver, and the last term is the fraction of T cells that bypasses the LN and exits at the efferent lymphatic vessel.

$$\frac{dT^{ins}_{LN\_Abd}}{dt}=\begin{pmatrix} P(A^{ins}_{LN\_Abd},T^{ins}_{LN\_Abd}) \\ +(1-ff_{liver})\ L_{liver}\ T^{ins}_{liver}\ fi_{t,liver}+(1-ff_{liver})\ L_{intestine}\ T^{ins}_{intestine}\ fi_{t,intestine} \\ +(1-ff_{liver})\ L_{spleen}\ T^{ins}_{spleen}\ fi_{t,spleen} \\ -\left((1-ff_{liver})L_{liver}+(1-ff_{liver})L_{intestine}+(1-ff_{liver})L_{spleen}+L_{LN\_Abd}\right) \\ \times(H(T^{th}_{LN\_Abd}-T^{ins}_{LN\_Abd})\ fi_{t,LN\_Abd}+H(T^{ins}_{LN\_Abd}-T^{th}_{LN\_Abd}))\,T^{ins}_{LN\_Abd} \end{pmatrix}/V^{ins}_{LN\_Abd} \tag{12b}$$

$$P(A^{ins}_{LN\_Abd},T^{ins}_{LN\_Abd})=\rho_{LN\_Abd}\ T^{ins}_{LN\_Abd}\ V^{ins}_{LN\_Abd}\ H_{(A^{ins}_{LN\_Abd}-A^{th})}\ H_{(T^{ins}_{LN\_Abd}+nTcell)}\ H_{(T^{th}_{LN\_Abd}-T^{ins}_{LN\_Abd})}$$

where the first term is the proliferation rate of T cells in antigen-positive LNs, the second, third and fourth terms are T cell flow from lymphatic flow of tissue into LNs, and the last term is the fraction of T cells that bypasses the LN and exits at the efferent lymphatic vessel. The proliferation rate of T cells is a function of antigen concentration in the LN, n T cell presence in the LN, and the level of effector T cells in the LN.

## d) Antigen distribution in LN of liver/spleen/intestines

$$\frac{dA^{b}_{LN\_Abd}}{dt}=\begin{pmatrix} (1-f_{liver})(Q_{intestine}-L_{intestine})\,A^{b}_{intestine} \\ +(1-f_{liver})(Q_{spleen}-L_{spleen})\,A^{b}_{spleen} \\ +(1-f_{liver})\ (Q_{liver}-Q_{intestine}-Q_{spleen}+L_{intestine}+L_{spleen})\,A^{a}_{Heart} \\ -\left((1-f_{liver})\ Q_{liver}-L_{LN\_Abd}\right)A^{b}_{LN\_Abd} \\ -J_{a,LN\_Abd}\ A^{b}_{LN\_Abd}\ V^{b}_{LN\_Abd} \end{pmatrix}/V^{b}_{LN\_Abd} \tag{13a}$$

where the first, second and third terms are the flow of antigen from the intestine, spleen and liver, to the LN; the fourth term is the flow of antigen in the exit blood stream, and the last term is the antigen that crosses the blood vessel to enter the LN. The mass balance for antigen within the liver/intestine/spleen draining lymph node tissue ($A^{ins}_{LNtumor}$) is:

$$\frac{dA^{ins}_{LN\_Abd}}{dt}=\begin{pmatrix} J_{a,LN\_Abd}\ A^{b}_{LN\_Abd}\ V^{b}_{LN\_Abd}+(1-ff_{liver})L_{liver}\ A^{ins}_{liver}\ fi_{a,liver} \\ (1-ff_{liver})L_{intestine}\ A^{ins}_{intestine}\ fi_{a,intestine}+(1-ff_{liver})L_{spleen}\ A^{ins}_{spleen}\ fi_{a,spleen} \\ -\left((1-ff_{liver})L_{liver}+(1-ff_{liver})L_{spleen}+(1-ff_{liver})L_{intestine}+L_{LN\_Abd}\right)A^{ins}_{LN\_Abd}\ fi_{a,LN\_Abd} \\ -\varepsilon_{LN\_Abd}\ A^{ins}_{LN\_Abd}\ V^{ins}_{LN\_Abd} \end{pmatrix}/V^{ins}_{LN\_Abd} \tag{13b}$$

where the first term is antigen entering the LN from the blood supply; the second term is the flow of antigen with the lymph via the afferent lymphatic vessel; the third term represents the fraction of antigen that bypasses the LN and exits at the efferent lymphatic vessel. The last term is the degradation of antigen in the node.

**Skin, Muscle, Bone, Brain and Kidney**

## a) T-cell trafficking in blood and interstitium of the *i*th organ, including skin, muscle, bone, brain and kidney:

$$\frac{dT_i^b}{dt}=\begin{pmatrix} f_i\ Q_i\ T_{Heart}^a-(f_i\ Q_i-L_i)T_i^b \\ -a_i\ T_i^b\ V_i^b \\ +d_i\ T_i^c\ V_i^b \end{pmatrix}/V_i^b \quad (14a)$$

where the first term is the flow of T cells from the arterial blood into the $i^{th}$ organ; the second term is the flow of T cells out of the $i^{th}$ organ; the third term represents the binding of T cells to the endothelium of $i^{th}$ organ, and the last term is the detachment of cells from the endothelium. In Eqs. 14b and c, the terms with $J$ are transmigration of T cells, and the term with $L$ is the outflow of T cells via lymphatic flow:

$$\frac{dT_i^c}{dt}=\left(a_i\ T_i^b\ V_i^b-d_i\ T_i^c\ V_i^b-J_{t,i}\ T_i^c\ V_i^b\right)/V_i^b \quad (14b)$$

$$\frac{dT_i^{ins}}{dt}=\left(J_{t,i}\ T_i^c\ V_i^b-L_i\ T_i^{ins}\ fi_{t,i}\right)/V_i^{ins} \quad (14c)$$

## b) Antigen distribution in blood vessels and interstitium

$$\frac{dA_i^b}{dt}=\left(f_i\ Q_i\ A_{Heart}^b-(f_i\ Q_i-L_i)\ A_i^b-J_{a,i}\ A_i^b\ V_i^b\right)/V_i^b \quad (15a)$$

$$\frac{dA_i^{ins}}{dt}=\left(J_{a,i}\ A_i^b\ V_i^b-L_i\ A_i^{ins}\ fi_{a,i}-\varepsilon_i\ A_i^{ins}V_i^{ins}\right)/V_i^{ins} \quad (15b)$$

where the first term of Eq. 15a is the flow of antigen from the arterial blood into the $i^{th}$ organ; the second term is the flow of antigen out of the $i^{th}$ organ via outlet venous blood flow; the third is transmigration of antigen. In Eq.15b, the second term is the outflow of antigen from interstitium and the last term is natural decay of antigen.

## c) T-cell trafficking in the LN of the ith compartment including skin, muscle, bone, brain and kidney:

$$\frac{dT_{LNi}^b}{dt}=\begin{pmatrix} (1-f_i)\ Q_i\ T_{Heart}^a \\ -\left((1-f_i)\ Q_i-L_{LNi}\right)T_{LNi}^b \\ \end{pmatrix}/V_{LNi}^b \quad (16a)$$

The first term is T cell flow from heart into LNs of $i^{th}$ organ and the second term is T cell leaving the LNs of $i^{th}$ organ via outlet venous blood flow,

$$\frac{dT_{LNi}^{ins}}{dt}=\begin{pmatrix} P(A_{LNi}^{ins},T_{LNi}^{ins}) \\ +(1-ff_i)\,L_i\,T_i^{ins}\;\;fi_{t,i} \\ -\big((1-ff_i)\,L_i+L_{LNi}\big) \\ \quad\times(H(T_{LNi}^{th}-T_{LNi}^{ins})\;fi_{t,LNi}+H(T_{LNi}^{ins}-T_{LNi}^{th}))\,T_{LNi}^{ins} \end{pmatrix}/V_{LNtumor}^{ins} \tag{16b}$$

$$P(A_{LNi}^{ins},T_{LNi}^{ins})=\rho_{LNi}\;T_{LNi}^{ins}\;V_{LNi}^{ins}\;H_{(A_{LNi}^{ins}-A^{th})}\;H_{(T_{LNi}^{ins}+nTcell)}\;H_{(T_{LNi}^{th}-T_{LNi}^{ins})}$$

Where the first term is the proliferation of T cells in the LNs of $i^{th}$ organ, the second term is T cells in the LNs from interstitial flow, the last term is T cell flow leaving the LNs via lymphatic flows.

**d) Antigen distribution in LN of compartment *i***

$$\frac{dA_{LNi}^{b}}{dt}=\begin{pmatrix} (1-f_i)\;Q_i\;\;A_{Heart}^{a} \\ -\big((1-f_i)\;Q_i-L_{LNi}\big)\,A_{LNi}^{b} \\ -J_{a,LNi}\;A_{LNi}^{b}\,V_{LNi}^{b} \end{pmatrix}/V_{LNi}^{b} \tag{17a}$$

$$\frac{dA_{LNi}^{ins}}{dt}=\begin{pmatrix} J_{a,LNi}\;A_{LNi}^{b}\,V_{LNi}^{b} \\ +(1-ff_i)\,L_i\;\;A_i^{ins}\;\;fi_{a,i} \\ -\big((1-ff_i)\,L_i+L_{LNi}\big)\,A_{LNi}^{ins}\;\;fi_{a,LNi} \\ -\varepsilon_{LNi}\;A_{LNi}^{ins}\;\;V_{LNi}^{ins} \end{pmatrix}/V_{LNi}^{ins} \tag{17b}$$

In Eq.17a, the first term is antigen flow from the heart into the $i^{th}$ organ, the second term is antigen out form the LNs, and the last term is transmigration. In Eq. 17b, the second term is antigen flow from interstitial flow, the third terms is outflow of antigen from the LNs to the circulation, and the last term is the natural decay of antigen.

## Heart

**a) T-cell recirculation in arterial and venous blood flows**

$$\frac{dT_{Heart}^{a}}{dt}=\begin{pmatrix} f_{lung}\,(Q_{lung}-L_{lung})\,T_{lung}^{b} \\ -(Q_{liver}+L_{spleen}+L_{\text{int}estine}+Q_{kidney}+Q_{tumor} \\ +Q_{skin}+Q_{muscle}+Q_{bone}+Q_{brain})\,T_{Heart}^{a} \end{pmatrix}/V_{Heart}^{a} \tag{18a}$$

Where the first term is the T cell flow from lung to left ventricle of heart and the second term is the T cell flow leaving the left ventricle to distribute among different organs.

$$\frac{dT^{v}_{Heart}}{dt}=\left(\begin{array}{l}\left((1-f_{lung})(Q_{lung}-L_{lung})-L_{LNlung}\right)T^{b}_{LNlung}\\ +(f_{tumor}\,Q_{tumor}-L_{tumor})T^{b}_{tumor}+\left((1-f_{tumor})\,Q_{tumor}-L_{LNtumor}\right)T^{b}_{LNtumor}\\ +(f_{liver}\,Q_{liver}-L_{liver})T^{b}_{liver}+\left((1-f_{liver})\,Q_{liver}-L_{LNliver}\right)T^{b}_{LNliver}\\ +(f_{i}\,Q_{i}-L_{i})T^{b}_{i}+\left((1-f_{i})\,Q_{i}-L_{LNi}\right)T^{b}_{LNi}\\ +\left((1-ff_{tumor})L_{tumor}+L_{LNtumor}\right)T^{ins}_{LNtumor}\;fi_{t,LNtumor}\\ +\left((1-ff_{lung})L_{lung}+L_{LNlung}\right)T^{ins}_{LNlung}\;fi_{t,LNlung}\\ +\left((1-ff_{liver})L_{liver}+(1-ff_{intestine})L_{intestine}+(1-ff_{spleen})L_{spleen}+L_{LNliver}\right)T^{ins}_{LNliver}\;fi_{t,LNliver}\\ +\left((1-ff_{i})L_{i}+L_{LNi}\right)T^{ins}_{LNi}\;fi_{t,LNi}\\ +\left(ff_{tumor}\,L_{tumor}\right)T^{ins}_{tumor}\;fi_{t,tumor}\\ +\left(ff_{lung}\,L_{lung}\right)T^{ins}_{lung}\;fi_{t,lung}\\ +\left(ff_{liver}\,L_{liver}\right)T^{ins}_{liver}\;fi_{t,liver}+\left(ff_{intestine}\,L_{intestine}\right)T^{ins}_{intestine}\;fi_{t,intestine}+\left(ff_{spleen}\,L_{spleen}\right)T^{ins}_{spleen}\;fi_{t,spleen}\\ +\left(ff_{i}\,L_{i}\right)T^{ins}_{i}\;fi_{t,i}\\ -Q_{lung}T^{v}_{Heart}\end{array}\right)/V^{v}_{Heart} \quad (18b)$$

Where all the positive terms are flow of T cells into venous flow of heart and the last term is collected T cell flow leaving the heart via right ventricle flow (inflow of lung).

**b) Arterial and venous recirculation of antigen**

$$\frac{dA^{a}_{Heart}}{dt}=\left(\begin{array}{l}f_{lung}\,(Q_{lung}-L_{lung})\,A^{b}_{lung}\\ -(Q_{liver}+L_{spleen}+L_{intestine}+Q_{kidney}+Q_{tumor}\\ +Q_{skin}+Q_{muscle}+Q_{bone}+Q_{brain})\,A^{a}_{Heart}\end{array}\right)/V^{a}_{Heart} \quad (19a)$$

Where the first term is the antigen flow from lung to left ventricle of heart and the second term is the antigen flow leaving the left ventricle to distribute among different organs.

$$\frac{dA^{v}_{Heart}}{dt}=\begin{pmatrix}\left((1-f_{lung})(Q_{lung}-L_{lung})-L_{LNlung}\right)A^{b}_{LNlung}\\+(f_{tumor}\,Q_{tumor}-L_{tumor})\,A^{b}_{tumor}+\left((1-f_{tumor})\,Q_{tumor}-L_{LNtumor}\right)A^{b}_{LNtumor}\\+(f_{liver}\,Q_{liver}-L_{liver})\,A^{b}_{liver}+\left((1-f_{liver})\,Q_{liver}-L_{LNliver}\right)A^{b}_{LNliver}\\+(f_{i}\,Q_{i}-L_{i})\,A^{b}_{i}+\left((1-f_{i})\,Q_{i}-L_{LNi}\right)A^{b}_{LNi}\\+\left((1-ff_{tumor})\,L_{tumor}+L_{LNtumor}\right)A^{ins}_{LNtumor}\;fi_{t,LNtumor}\\+\left((1-ff_{lung})L_{lung}+L_{LNlung}\right)A^{ins}_{LNlung}\;fi_{t,LNlung}\\+\left((1-ff_{liver})L_{liver}+(1-ff_{\text{int}estine})L_{\text{int}estine}+(1-ff_{spleen})L_{spleen}+L_{LNliver}\right)A^{ins}_{LNliver}\;fi_{t,LNliver}\\+\left((1-ff_{i})\,L_{i}+L_{LNi}\right)A^{ins}_{LNi}\;fi_{t,LNi}\\+\left(ff_{tumor}\,L_{tumor}\right)A^{ins}_{tumor}\;fi_{t,tumor}\\+\left(ff_{lung}\,L_{lung}\right)A^{ins}_{lung}\;fi_{t,lung}\\+\left(ff_{liver}\,L_{liver}\right)A^{ins}_{liver}\;fi_{t,liver}+\left(ff_{\text{int}estine}\,L_{\text{int}estine}\right)A^{ins}_{\text{int}estine}\;fi_{t,\text{int}estine}+\left(ff_{spleen}\,L_{spleen}\right)A^{ins}_{spleen}\;fi_{t,spleen}\\+\left(ff_{i}\,L_{i}\right)A^{ins}_{i}\;fi_{t,i}\\-Q_{lung}\,A^{v}_{Heart}\end{pmatrix}/V^{v}_{Heart}\quad(19b)$$

Where all the positive terms are the flows of antigen into venous flow of heart and the last term is the collected antigen flow leaving the heart via right ventricle flow (inflow of lung).

## c) Mass conservation equations

$$\begin{aligned}f_{lung}\;\;Q_{lung}=\;&f_{lung}\;\;L_{lung}+Q_{heart-to-liver}+Q_{spleen}+Q_{\text{int}estine}+Q_{kidney}+Q_{tumor}\\&+Q_{skin}+Q_{muscle}+Q_{bone}+Q_{brain}\end{aligned}\quad(20a)$$

$$Q_{liver}\;=Q_{heart-to-liver}+(Q_{spleen}-L_{spleen})+(Q_{\text{int}estine}-L_{\text{int}estine})\quad(20b)$$

## Model validation

To compare the model predictions with clinical observations, we simulated radiation therapy (RT) for six patients and measured tumor growth (Fig. 9S). Figs. 9S A-C are lung cancer patients treated with RT (2Gy, administered every day except weekends), and Figs. 9S D-F show cervical cancer patients with the same RT schedule. For each patient, the growth rate of tumor and the constant values of the linear-quadratic model of RT are assigned based on the experimental data. According to these results, the model can reproduce the tumor size reduction of different patients treated with RT, if we use the correct patient-specific parameters.

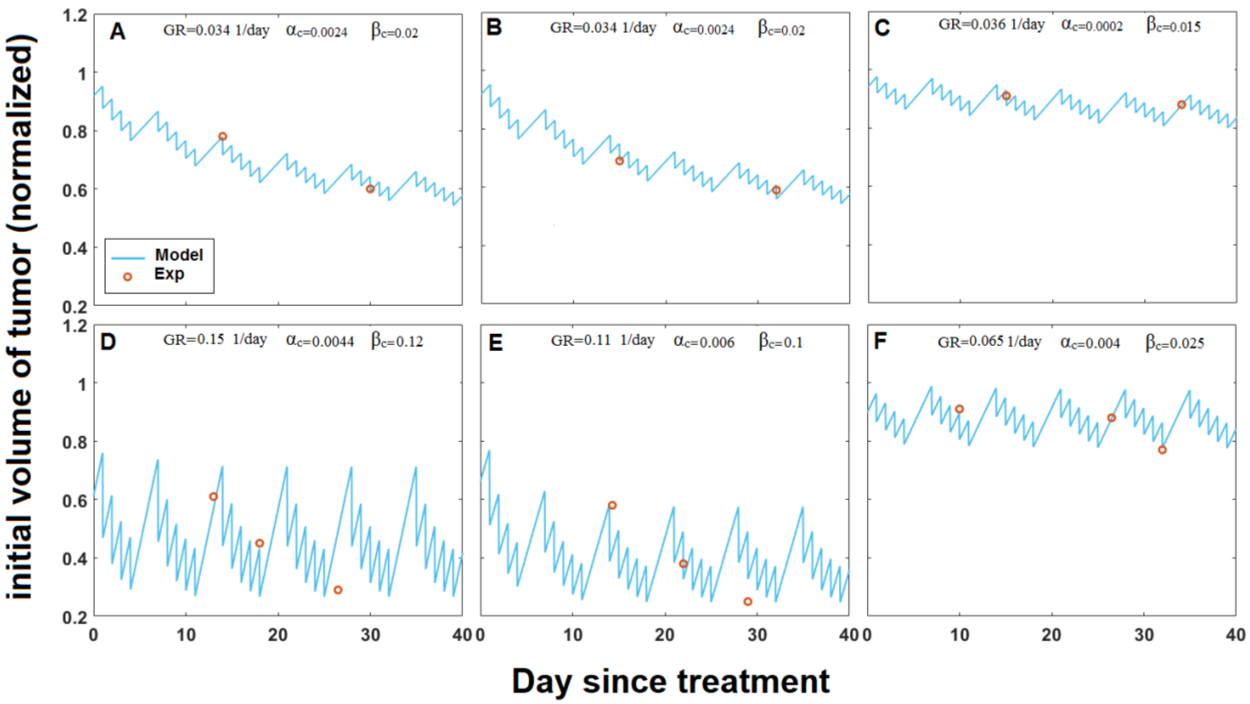


**Figure S9.** Comparison between model prediction and clinical results of tumor size reduction by RT with 2 Gy, injected daily for 5 days a week, A-B) lung cancer patients under RT with 2Gy, D-F) cervical cancer patients under RT with 2Gy.

## Table S1. Model parameters

| T-cell Transvascular | Description | constant | Ref |
|---|---|---|---|
| $J_{t,tumor}$ | Transvascular migration rate of captured T cell into tumor tissue | $2.1\times10^{-4}$ min$^{-1}$ | [4,5] |
| $J_{t,LNtumor}$ | Transvascular migration rate of captured T cell into tumor LN | $2.1\times10^{-4}$ min$^{-1}$ | [4,5] |
| $J_{t,i}$ | Transvascular migration rate of captured T cell into i$^{th}$ tissues; i = lung, liver, intestine, spleen, and the rest of $i^{th}$ tissues | $2.1\times10^{-4}$ min$^{-1}$ | [4,5] |
| **Antigen Transvascular** | **Description** | **constant** | **Ref** |
| $J_{a,tumor}$ | Transvascular filtration rate of antigen into tumor tissue | 0.43 1/d | Estimated |
| $J_{a,LNtumor}$ | Transvascular filtration rate of antigen into tumor LN | 0.21 1/d | Estimated |
| $J_{a,i}$ | Transvascular filtration rate of antigen into i$^{th}$ tissues; i = lung, liver, intestine, spleen, and the rest of $i^{th}$ tissues | 0.114 1/d | Estimated |
| $J_{a,LNi}$ | Transvascular filtration rate of antigen into i$^{th}$ LNs; i = lung, abdominal, and the rest of $i^{th}$ LNs | 0.114 1/d | Estimated |
| **T cell adhesion** | **Description** | **constant** | **Ref** |
| $a_{tumor}$ | Constant attachment rate constant of T cell to the blood vessel wall of tumor ; Variable attachment rate of tumor: $a'_{tumor} = a_{tumor}(V/(1+V))$ , $V$ : *tumor volume* | $6.9\times10^{-3}$ min$^{-1}$ | [4] |
| $a_{lung}$ | Attachment rate of T cell to the blood vessel wall in lung | $3.1\times10^{-6}$ min$^{-1}$ | [4] |

| | | | |
|---|---|---|---|
| $a_{liver}$ | Attachment rate of T cell to the blood vessel wall in liver | $7.6\times10^{-9}$ min$^{-1}$ | [4] |
| $a_{spleen}$ | Attachment rate of T cell to the blood vessel wall in spleen | $8.1\times10^{-6}$ min$^{-1}$ | [4] |
| $a_{intestine}$ | Attachment rate of T cell to the blood vessel wall in intestine | $8.1\times10^{-6}$ min$^{-1}$ | [4] |
| $d_j$ | detachment rate of T cell from the blood vessel wall of all tissues | 200 min$^{-1}$ | [6,7] |
| **Antigen natural decay** | **Description** | **constant** | **Ref** |
| $\varepsilon_{tumor}$ | Antigen natural decay rate in tissues and LNs interstitium | 0.015 1/d | Estimated |
| **Blood flow rate** | **Description** | **constant** | **Ref** |
| $Q_{tumor}$ | Initial Blood flow rate of tumor | 0.564 [ml/min] | [4] |
| $Q_{skin}$ | Blood flow rate of skin | 220 [ml/min] | [4] |
| $Q_{muscle}$ | Blood flow rate of muscle | 413 [ml/min] | [4] |
| $Q_{bone}$ | Blood flow rate of bone | 138 [ml/min] | [8] |
| $Q_{liver}$ | Blood flow rate of liver (hepatic portal vein from G.I. and spleen, and hepatic artery) | 800 [ml/min] | [8] |
| $Q_{spleen}$ | Blood flow rate of spleen | 138 [ml/min] | [8] |
| $Q_{intestine}$ | Blood flow rate of intestine | 468 [ml/min] | [8] |
| $Q_{kidney}$ | Blood flow rate of kidney | 630 [ml/min] | [8] |
| $Q_{brain}$ | Blood flow rate of brain | 300 [ml/min] | Estimate - [8] |
| $Q_{cardic\ vessels}$ | Blood flow rate of cardiac vessels | 120 [ml/min] | Estimate - [8] |
| **Lymphatic flow rate** | **Description** | **constant** | **Ref** |
| $L_{tumor}$ | Lymphatic flow rate of tumor | 3x$10^{-2}$ [ml/min] | [4] |
| $L_{liver}$ | Lymphatic flow rate of liver | 8.7x$10^{-2}$ [ml/min] | [8] |
| $L_{spleen}$ | Lymphatic flow rate of spleen | 8.7x$10^{-4}$ [ml/min] | [8] |
| $L_{intestine}$ | Lymphatic flow rate of intestine | 3.0 x $10^{-1}$ [ml/min] | [8] |
| $L_{kidney}$ | Lymphatic flow rate of kidney | 1x $10^{-3}$ [ml/min] | Estimate - [8] |
| $L_{brain}$ | Lymphatic flow rate of brain | 1x $10^{-3}$ [ml/min] | Estimate - [8] |
| $L_{cardic\ vessels}$ | Lymphatic flow rate of cardiac vessels | 4.3x $10^{-3}$ [ml/min] | Estimate - [8] |
| $L_{Hlung}$ | Lymphatic flow rate of lung | 4.3x $10^{-2}$ [ml/min] | [8] |
| $L_{LNj}$ | Lymphatic flow rate of LNs | 7.7x$10^{-1}$ [ml/min] | [4] |
| **Blood and lymphatic flow fraction** | **Description** | **constant** | **Ref** |
| $f_j$ | Fractional flow rate of blood into tumor tissue | 0.995, 0.99, 0.95, 0.9 | Estimated |

| | | | |
|---|---|---|---|
| $ff_j$ | Fractional rate of lymphatic flow from tissue part to the main lymphatic flow | 0.99 | Estimated |
| T cell fraction from interstitium to lymphatic flow | Description | constant | Ref |
| $f_{t,j}$ | T cell fraction from tissue interstitium of all compartments except tumor to lymphatic flow | 1 | 9 |
| $f_{t,tumor}$ | T cell fraction from tissue interstitium of tumor to lymphatic flow | 0 | 9 |
| $f_{t,LNj}$ | T cell fraction from LNs interstitium to lymphatic flow | 0.018 | 9 |
| Antigen fraction from interstitium to lymphatic flow | Description | constant | Ref |
| $f_{a,j}$ | Antigen fraction from tissue interstitium of all compartments to lymphatic flow | 1 | Estimate-[9] |
| $f_{a,LNj}$ | Antigen fraction from LNs interstitium to lymphatic flow | 0.18 | Estimate-[9] |
| Volume of tissue interstitium | Description | constant | Ref |
| $V^{ins}_{tumor}$ | Averaged interstitium volume of tumor | 10.9 [ml] | 4 |
| $V^{ins}_{liver}$ | Averaged interstitium volume of liver | 361.8 [ml] | 8 |
| $V^{ins}_{spleen}$ | Averaged interstitium volume of spleen | 34.7 [ml] | 8 |
| $V^{ins}_{intestine}$ | Averaged interstitium volume of intestine | 373.2 [ml] | 8 |
| $V^{ins}_{kidney}$ | Averaged interstitium volume of kidney | 96.6 [ml] | 8 |
| $V^{ins}_{brain}$ | Averaged interstitium volume of brain | 279 [ml] | Estimate |
| $V^{ins}_{cardic\ vessels}$ | Averaged interstitium volume of cardiac vessels | 42.9 [ml] | Estimate |
| $V^{ins}_{lung}$ | Averaged interstitium volume of normal part of lung | 299.7 [ml] | 8 |
| $V^{ins}_{bone}$ | Averaged interstitium volume of bone | 279 [ml] | 8 |
| $V^{ins}_{muscle}$ | Averaged interstitium volume of muscle | 4558 [ml] | 8 |
| $V^{ins}_{skin}$ | Averaged interstitium volume of skin | 227 [ml] | 8 |
| Volume of LN interstitium | Description | constant | Ref |
| $V^{ins}_{LNs}$ | Averaged interstitium volume of LNs | 34.7 [ml] | 4 |
| Volume of vascular tissue | Description | constant | Ref |
| $V^{b}_{tumor}$ | Averaged vascular volume of tumor | 1.4 [ml] | 4 |
| $V^{b}_{liver}$ | Averaged vascular volume of liver | 180.9 [ml] | 8 |
| $V^{b}_{spleen}$ | Averaged vascular volume of spleen | 17 [ml] | 8 |
| $V^{b}_{intestine}$ | Averaged vascular volume of intestine | 43 [ml] | 8 |
| $V^{b}_{kidney}$ | Averaged vascular volume of kidney | 28.4 [ml] | 8 |
| $V^{b}_{brain}$ | Averaged vascular volume of brain | 150 [ml] | Estimate |
| $V^{a}_{Heart}$ | Averaged volume of arterial cardiac vessels | 69.9 [ml] | Estimate |

| | | | |
|---|---|---|---|
| $V^{v}_{Heart}$ | Averaged volume of venus cardiac vessels | 60.6 [ml] | Estimate |
| $V^{b}_{lung}$ | Averaged vascular volume of normal part of lung | 99.9 [ml] | 8 |
| $V^{b}_{bone}$ | Averaged vascular volume of bone | 150 [ml] | 8 |
| $V^{b}_{muscle}$ | Averaged vascular volume of muscle | 700 [ml] | 8 |
| $V^{b}_{skin}$ | Averaged vascular volume of skin | 462 [ml] | 8 |
| **Volume of vascular LN** | **Description** | **constant** | **Ref** |
| $V^{b}_{LNtumor}$ | Averaged vascular volume of LNs | 17 [ml] | 4 |
| **Antigen-induced T cell proliferation** | **Description** | **constant** | **Ref** |
| $\rho_{LNj}$ | T cell proliferation rate in LNs | $1.4 \times 10^{-3}$ min$^{-1}$ | Estimated |
| $A^{th}$ | Minimum antigen concentration for T cell proliferation | $1.4 \times 10^{-3}$ [1/ml] | Estimated |
| $T^{th}_{LNj}$ | T cell capacity of LNs | $1 \times 10^{3}$ [1/ml] | Estimated |
| $a_c$ | Production rate of antigen by cancer cells | Low-antigen: $1 \times 10^{-5}$ min$^{-1}$<br>High-antigen: $7 \times 10^{-3}$ min$^{-1}$ | Estimated |
| $a_{c,I}$ | Production rate of antigen by immune-induced apoptotic cancer cells | Low-antigen: $3 \times 10^{-5}$ min$^{-1}$<br>High-antigen: $2.1 \times 10^{-2}$ min$^{-1}$ | Estimated |
| $a_{c,RT}$ | Production rate of antigen by radiation-induced apoptotic cancer cells | Low-antigen: $3 \times 10^{-3}$ min$^{-1}$<br>High-antigen: $2.1 \times 10^{-2}$ min$^{-1}$ | Estimated |
| **Radiotherapy model** | **Description** | **constant** | **Ref** |
| $\xi_c$ | Scale factor for the radiosensitivity of cancer cells | 1 | 10 |
| $\alpha_c$ | cell type-specific radiosensitivity parameters for cancer cells | 0.3 | 10 |
| $\beta_c$ | cell type-specific radiosensitivity parameters for cancer cells | 0.03 | 10 |
| $\xi_v$ | Scale factor for the radiosensitivity of endothelial cells | 1 | 10 |
| $\alpha_v$ | cell type-specific radiosensitivity parameters for endothelial cells | 0.215 | 10 |
| $\beta_v$ | cell type-specific radiosensitivity parameters for endothelial cells | 0.028 | 10 |
| $\xi_T$ | Scale factor for the radiosensitivity of T cells | 1 | 10 |
| $\alpha_T$ | cell type-specific radiosensitivity parameters for T cells | 0.205 | 10 |
| $\beta_T$ | cell type-specific radiosensitivity parameters for T cells | 0.025 | 10 |
| $\varepsilon_{ap,RT}$ | Decay rate of RT-killed cancer, vessels, and T cells | $1 \times 10^{-3}$ min$^{-1}$ | Estimated |
| **Tumor-immune interaction** | **Description** | **constant** | **Ref** |
| $k_1$ | T cell /tumor cell complex formation rate | | 11 |

| | | | |
|---|---|---|---|
| | | 1.3 ×$10^{-7}$ $day^{-1}$ $(cell/ml)^{-1}$ | |
| $k_{-1}$ | T cell /tumor cell complex degeneration rate | 24 $day^{-1}$ | [11] |
| $k_2$ | T cell/tumor cell complex stabilization rate | 7.2 $day^{-1}$ | [11] |
| $p_r$ | PD-L1/PD-1 blockade efficiency | 0.9997 | Estimated- [11] |
| $\beta_v$ | maximum value to enhance the accessibility of T cells to tumor cells by angiogenic vessels | 10 | Estimated- [11] |
| $\alpha_v$ | M-M constant to enhance the accessibility of T cells to tumor cells by angiogenic vessels | 1 | [11] |
| **Tumor growth and Angiogenesis** | **Description** | **constant** | **Ref** |
| $g_v$ | Maximum rate of vascular tumor growth | 1.4×$10^{-3}$ $min^{-1}$ | Estimated - [12,13] |
| $g_{av}$ | Maximum rate of avascular tumor growth | 7×$10^{-7}$ $min^{-1}$ | Estimated - [12,13] |
| $\lambda_{a0}$ | M-M constant for Maximum avascular tumor growth | 1 | Estimated - [12,13] |
| $\eta_{ag}$ | Production rate of tumor-induced VEGF | 1.1×$10^{-10}$ $min^{-1}$ | Estimated- [14,15] |
| $\varepsilon_{vegf}$ | Natural decay rate of tumor-induced VEGF | 7×$10^{-5}$ $min^{-1}$ | Estimated- [14,15] |
| $\beta_{ag}$ | Maximum rate of angiogenesis | 1.4×$10^{-4}$ $min^{-1}$ | Estimated- [14,15] |
| $\alpha_{vegf0}$ | M-M constant of angiogenesis rate | 1×$10^{-6}$ | Estimated- [14,15] |
| $\omega_{ag}$ | Maximum value of angiogenic vessel degradation | 3.3×$10^{-3}$ $min^{-1}$ | Estimated- [14,15] |
| $\theta_{vegf0}$ | M-M constant of angiogenic vessel degradation | 3.3×$10^{-7}$ | Estimated- [14,15] |
| **residence time** | **Description** | **constant** | **Ref** |
| $tr_{LN}$ | Residence time of n T cells in LNs | 10 hrs | [1,16] |
| $tr_{spleen}$ | Residence time of n T cells in spleen | 2.5 hrs | [1,16] |
| $tr_{lung}$ | Residence time of n T cells in lung | 0.46 min | [1,16] |
| $tr_{liver}$ | Residence time of n T cells in liver | 0.88 min | [1,16] |
| $tr_i$ | Residence time of n T cells in other organs | 0.21 min | Estimated,[1,16] |

## References

1 Ganusov, V. V. & Auerbach, J. Mathematical modeling reveals kinetics of lymphocyte recirculation in the whole organism. *PLoS computational biology* **10**, e1003586 (2014).

2 du Bois, H., Heim, T. A. & Lund, A. W. Tumor-draining lymph nodes: At the crossroads of metastasis and immunity. *Science immunology* **6**, eabg3551 (2021).

3 McMahon, S. J. The linear quadratic model: usage, interpretation and challenges. *Physics in Medicine & Biology* **64**, 01TR01 (2018).

4 Zhu, H., Melder, R. J., Baxter, L. T. & Jain, R. K. Physiologically based kinetic model of effector cell biodistribution in mammals: implications for adoptive immunotherapy. *Cancer research* **56**, 3771-3781 (1996).

5 Friedrich, S. W. *et al.* Antibody-directed effector cell therapy of tumors: analysis and optimization using a physiologically based pharmacokinetic model. *Neoplasia* **4**, 449-463 (2002).

6 Melder, R. J., Salehi, H. A. & Jain, R. K. Interaction of activated natural killer cells with normal and tumor vessels in cranial windows in mice. *Microvascular research* **50**, 35-44 (1995).

7 Bjerknes, M., Cheng, H. & Ottaway, C. A. Dynamics of lymphocyte-endothelial interactions in vivo. *Science* **231**, 402-405 (1986).

8 Zhu, H., Melder, R. J., Baxter, L. T. & Jain, R. K. Physiologically based kinetic model of effector cell biodistribution in mammals: implications for adoptive immunotherapy. *Cancer Res* **56**, 3771-3781 (1996).

9 Butcher, E. Following cellular traffic: methods of labelling lymphocytes and other cells to trace their migration in vivo. *Handbook of experimental immunology* **2**, 57.51 (1986).

10 Alfonso, J. C. *et al.* Tumor-immune ecosystem dynamics define an individual Radiation Immune Score to predict pan-cancer radiocurability. *Neoplasia* **23**, 1110-1122 (2021).

11 Matzavinos, A., Chaplain, M. A. & Kuznetsov, V. A. Mathematical modelling of the spatio-temporal response of cytotoxic T-lymphocytes to a solid tumour. *Mathematical Medicine and Biology* **21**, 1-34 (2004).

12 Watanabe, Y., Dahlman, E. L., Leder, K. Z. & Hui, S. K. A mathematical model of tumor growth and its response to single irradiation. *Theoretical Biology and Medical Modelling* **13**, 1-20 (2016).

13 Enderling, H. & AJ Chaplain, M. Mathematical modeling of tumor growth and treatment. *Current pharmaceutical design* **20**, 4934-4940 (2014).

14 Nikmaneshi, M. R., Firoozabadi, B., Mozafari, A. & Munn, L. L. A multi-scale model for determining the effects of pathophysiology and metabolic disorders on tumor growth. *Scientific reports* **10**, 1-20 (2020).

15 Nikmaneshi, M. R., Firoozabadi, B. & Mozafari, A. Chemo-Mechanistic multi-scale model of a three-dimensional tumor microenvironment to quantify chemotherapy response of cancer. *Biotechnology and Bioengineering* (2021).

16 Breart, B. & Bousso, P. S1P1 downregulation tailors CD8+ T-cell residence time in lymph nodes to the strength of the antigenic stimulation. *European Journal of Immunology* **46**, 2730-2736 (2016).
